# Deep Learning Prediction of *O*‐Glycopeptide Tandem Mass Spectra Enhances *O*‐Glycoproteomics

**DOI:** 10.1002/advs.76760

**Published:** 2026-07-23

**Authors:** Yu Zong, Yuxin Wang, Liang Qiao

**Affiliations:** ^1^ Department of Chemistry, and Minhang Hospital Fudan University Shanghai China; ^2^ Cancer Science Institute of Singapore National University of Singapore Singapore Singapore; ^3^ Department of Computer Science, and Institute of Modern Languages and Linguistics Fudan University Shanghai China

**Keywords:** deep learning, graph neural network, mass spectrometry, O‐glycoproteomics, O‐glycosite localization

## Abstract

Protein glycosylation, a post‐translational modification involving the attachment of glycans to proteins, plays critical roles in numerous physiological and pathological cellular functions. Characterization of protein glycosylation is one of the most challenging problems due to the high heterogeneity of glycosites and glycan structures. Recently, deep learning has been adopted to predict *N*‐glycopeptide tandem mass spectrometry (MS/MS) spectra and exhibited a promising effect in *N*‐glycoproteomics analysis. However, current deep learning frameworks struggle to accurately predict *O*‐glycopeptide MS/MS spectra due to the complexity of *O*‐glycopeptides and the limited availability of training data. In this study, we introduce DeepGPO, a deep learning framework for the prediction of *O*‐glycopeptide MS/MS spectra. The DeepGPO incorporates a Transformer module alongside two graph neural network modules designed for handling branched glycans. To address the issue of data scarcity in *O*‐glycoproteomics, various training methods are adopted in DeepGPO, such as the introduction of training weights for different MS/MS spectra and the adoption of pre‐training strategies. With the predicted MS/MS, *O*‐glycosylation sites can be localized even in the absence of site‐determining ions. Currently, DeepGPO supports both mono‐ and double‐*O*‐glycosylated peptides. It shows promising application in clinical human *O*‐glycoproteomics. We anticipate that DeepGPO will inspire future advancements in glycoproteomics research.

## Graphical abstract

DeepGPO integrates Transformer and graph neural networks with tailored training strategies, including data augmentation, loss re‐weighting, and pre‐training, to achieve high‐quality *O*‐glycopeptide MS/MS spectra prediction. The predicted MS/MS spectra are used to localize *O*‐glycosylation sites from HCD MS/MS data, enhancing *O*‐glycoproteomics analysis and profiling site‐ specific tumor‐associated glycosylation changes.

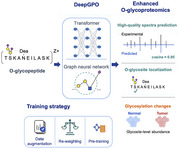

## 1 Introduction

Glycosylation is one of the most significant post‐translational modifications (PTMs) of proteins, playing a crucial role in numerous physiological and pathological cellular functions [1, 2]. In‐depth research into glycosylation is critical in medical treatment, diagnosis and prognosis [3]. Glycosylation is highly complex, characterized by dynamically modified amino acids, varied monosaccharide compositions, and intricate glycan structures. In proteins, glycans are usually attached to the side chains of asparagine (*N*‐glycosylation) or serine/threonine (*O*‐glycosylation). Despite the complexity of the branched structures of glycans for both *N*‐ and *O*‐glycosylation, *O*‐glycosylation presents even more significant analytical challenges because there is no specific *O*‐glycosylation motif and many serine/threonine can be potentially modified [4]. These heterogeneities pose substantial challenges in distinguishing glycopeptide isomers [5, 6].

To date, liquid chromatography coupled to tandem mass spectrometry (LC‐MS/MS) is the leading method in glycoproteomics research. Tandem mass spectrometry (MS/MS) is used to characterize glycopeptides based on accurate precursor masses and specific fragment ions. One major challenge in glycoproteomics, particularly in *O*‐glycoproteomics, is glycosite localization. Search engines, such as pGlyco3 [7], MSFragger‐Glyco [8] and MetaMorpheus [9], typically rely on site‐determining ions for glycosite localization, using MS/MS spectra generated from electron transfer dissociation (ETD), which can preserve glycan modifications during fragmentation. However, the efficiency of electron‐based dissociation depends heavily on precursor ion charge density and often fails to produce sufficient fragments. ETD is also a relatively slow procedure, limiting the analysis throughput. Collision‐induced dissociation methods, such as higher‐energy collisional dissociation (HCD), are fast and more efficient in fragmentation, but often lead to the loss of glycan modifications during fragmentation, generating MS/MS spectra missing site‐specific ions, which cannot be efficiently used for *O*‐glycosite localization. Alternatively, *O*‐glycoproteases, such as OgpA [10, 11], IMPa [12] and StcE [13], can cleave proteins adjacent to glycosylated residues and are therefore used for O‐glycosylation site identification. However, the specificity and cleavage efficiency of many enzymes remain suboptimal [4].

Unlike sequence searching based methods, spectra‐match is used for peptide identification by matching experimental MS/MS spectra with reference spectra, utilizing both intensity and m/z information. This approach overcomes the need for the observation of site‐determining fragment ions for glycosite localization and improves identification sensitivity [14]. However, constructing glycopeptide MS/MS spectral library remains challenging due to the difficulty of glycopeptide synthesis and the complexity of biological samples. As an alternative, deep‐learning prediction of peptide MS/MS spectra offers an efficient method for constructing spectral libraries. Various deep learning‐based MS/MS spectra prediction tools, such as pDeep series [15, 16, 17], Prosit [18], DeepMass:Prism [19], DeepDIA [20], DeepPhospho [21], DeepFLR [22], DeepGlyco [23] and DeepGP [24], have been developed. DeepGlyco [23] and DeepGP [24] are recently published deep‐learning models for *N*‐glycopeptides MS/MS spectra prediction. Despite these advancements, none of the tools can predict MS/MS spectra for *O*‐glycopeptides to date.

Herein, we present a deep‐learning based framework, DeepGPO, for *O*‐glycopeptides MS/MS spectra prediction. DeepGPO is composed of a Transformer module alongside two graph neural network (GNN) modules specifically designed for handling branched glycans. Advanced training techniques are employed to mitigate the limited availability of *O*‐glycopeptides training data. DeepGPO has explored the potential of spectral searching for *O*‐glycopeptides and realized *O*‐glycosite localization based on HCD MS/MS spectra, which normally contain insufficient site‐specific ions.

## 2 Results

### 2.1 DeepGPO Framework and Training Strategy

**FIGURE 1:**
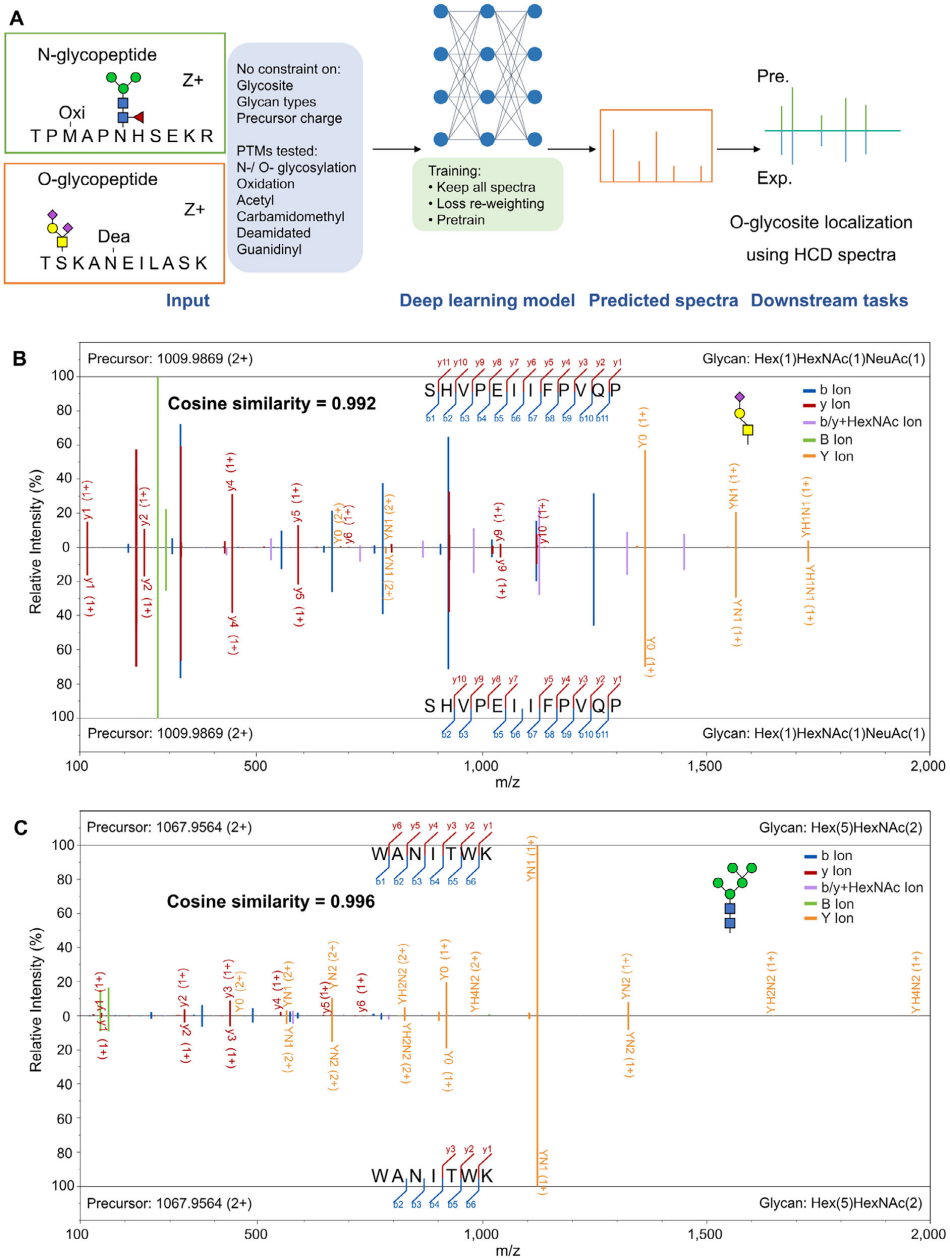
DeepGPO framework and glycopeptide MS/MS spectra prediction. (A) The framework of DeepGPO. Comparison of the experimental and predicted MS/MS spectra of (B) an *O*‐glycopeptide and (C) an *N*‐glycopeptide. Top: the predicted MS/MS spectrum; Bottom: the experimental MS/MS spectrum.

DeepGPO is based on our recently published DeepGP model for *N*‐glycopeptides [24]. The deep learning model of DeepGPO incorporates a Transformer module alongside two GNN modules for handling branched glycans (Figure S1). DeepGPO is capable of predicting both *N*‐ and *O*‐glycopeptides MS/MS spectra (Figure 1A). B/Y and b/y ions are considered concurrently to preserve the relative intensity of the fragment ions. Up to 16 types of B/Y fragment ions or 24 types of b/y fragment ions are considered for each cleavage event by combining various neutral losses and charge states (see details in Materials and Methods). To improve DeepGPO's generalization across *N*‐ and *O*‐glycopeptides, no restrictions are imposed on glycosites or glycan types. Besides, DeepGPO considers the common modifications, including Oxidation [M], Acetyl [Protein N‐term] and Carbamidomethyl [C], and two other modifications often encountered in *O*‐glycoproteomics, namely Deamidated [N] and Guanidinyl [K]. Deamidation occurs when PNGase F is used to remove *N*‐glycosylation, and guanidination of lysine is frequently applied to prevent conjugation of lysine residues to aldehyde groups and to enable the identification of glycopeptides containing lysine residues [11]. DeepGPO supports any possible glycopeptide length and precursor charge state. These features make DeepGPO a highly versatile deep learning‐based pipeline for glycopeptides analysis.

In this work, HCD MS/MS spectra of *O*‐glycopeptides serve as the training, validation and test data to explore the potential of HCD in *O*‐glycoproteomics due to its advantages in speed and fragmentation efficiency. Nevertheless, to obtain the *O*‐glycosite localization information by database searching using the state‐of‐the‐art search engines, HCD‐pd‐EThCD datasets were collected, wherein DeepGPO utilizes the parent HCD MS/MS spectra for training/validation/test, while glycopeptide identification is performed by pGlyco3 using the parent HCD and the triggered EThCD MS/MS spectra. DeepGPO adopts sqrt‐cosine similarity as the primary metric for glycopeptide MS/MS prediction during model training, with the consideration that peak intensities follow a Poisson distribution and that the square root transformation can stabilize the peak intensity variance [25]. This transformation is particularly advantageous as it assigns greater weight to low‐intensity peaks, thereby enhancing the prediction accuracy for low‐abundance ions. DeepGPO incorporates any peak identified as a fragment of glycopeptide into its metric calculation, not just those predicted by DeepGPO. Beyond sqrt‐cosine, DeepGPO also employs additional metrics for performance presentation, including cosine similarity (COS) and Pearson correlation coefficient (PCC).

Given the substantial scarcity of *O*‐glycoproteomics datasets and the complexity of *O*‐glycopeptides, we implemented three strategies to optimize DeepGPO's performance. First, we retain all glycopeptide MS/MS spectra during model training without removing duplicates. This approach acts as a form of data augmentation, where the intrinsic variations in different MS/MS spectra for the same glycopeptide replace manual augmentation methods.

Second, we introduce a loss re‐weighting method. Training weights for glycopeptide MS/MS spectra are assigned based on pGlyco3 identification results and *O*‐glycoprotease specificity, as illustrated in Figure S2. Briefly, an initial weight is derived from the pGlyco3 reported glycosite localization probability, which reflects the confidence of the identification. This weight is then further refined according to several criteria: (1) If the reported glycosite is inconsistent with the LocalizedSiteGroup by pGlyco3, or if the localization probability is unavailable, the weight is set to 0; (2) For peptides with multiple candidate sites identified by pGlyco3, the weight is adjusted according to the distribution of probabilities among the sites; (3) When *O*‐glycoprotease digestion is applied, identifications consistent with the enzyme specificity are doubled, with a minimum of 1; (4) Glycopeptides with only a single possible glycosylation site are assigned with a weight of 1. A higher weight indicates a higher confidence in the identification result. During model training, only MS/MS spectra with weights exceeding 0.5 are considered, and these weights are integrated into the loss function by multiplying the loss function by the corresponding weight for each MS/MS spectrum. As a result, inaccurate predictions for MS/MS spectra with higher weights incur a greater penalty for DeepGPO. This design follows the general principle of importance‐weighted learning [26], in which samples are assigned different contributions according to their reliability or relevance. The loss re‐weighting strategy enables DeepGPO to benefit from a large and diverse training set while reducing the influence of potentially noisy labels. To further evaluate the contribution of the strategy, we performed an ablation experiment by assigning an equal weight of 1 to all the MS/MS spectra. As shown in Figure S3, in later epochs, the model trained with confidence‐based re‐weighting reached a higher performance plateau, demonstrating that the loss re‐weighting strategy improves the final model performance. Critically, we ensure no glycopeptides overlap between the training and validation/test datasets. It is noted that the strategy is not restricted to the search results of pGlyco3. It can also be applied to other search engines by replacing the pGlyco3 probability score with other scores normalized to the range of 0 to 1.

Furthermore, a pre‐training strategy is implemented. We have trained DeepGPO based on Transformer without pre‐training [27], BERT [28] and DeepGP [24]. These models are of the same model configuration, but vary on the usage of pre‐training. Transformer starts with randomly initialized parameters. BERT benefits from pre‐training with unlabeled natural language. DeepGP has been trained using *N*‐glycopeptides MS/MS spectra. The benchmarking dataset (Dataset 1) comprises 18,260 MS/MS spectra encompassing 2,730 unique *O*‐glycopeptides with the weight exceeding 0.5 (PXD037415, Table S1, Note S1) [29]. The dataset was divided into the training and validation subset at a 9:1 ratio. The model's performance was evaluated on the validation set during training using different base models. The result showed that DeepGPO built upon DeepGP achieved the highest median sqrt‐cosine similarity in predicting *O*‐glycopeptide MS/MS spectra while requiring the fewest training epochs (Figure S4), indicating that the pre‐training strategy could benefit downstream tasks. Multi‐stage fine‐tuning could further enhance the performance. Using the model trained by other *O*‐glycoproteomics datasets (Dataset 2–11 [10, 11, 30, 31, 32, 33, 34, 35, 36], Table S1) as the base model, DeepGPO can achieve better performance on the same validation data from Dataset 1 (Figure S5).

The prediction performance using one *O*‐glycopeptide and one *N*‐glycopeptide as examples is shown (Figure 1B,C). The two glycopeptides are both from Dataset 1 but with different experimental conditions (Note S1). The *N*‐glycopeptide spectrum is acquired by sceHCD and the *O*‐glycopeptide spectrum is the parent HCD MS/MS spectrum from the HCD‐pd‐EThCD MS/MS spectrum. Due to the different experimental conditions of *N*‐ and *O*‐glycopeptides MS/MS spectra, DeepGPO was trained separately. The model for the prediction of the *O*‐glycopeptide MS/MS spectra was the Finetuned‐DeepGPO model in Figure S5. For the prediction of the *N*‐glycopeptide MS/MS spectra, DeepGPO was trained with 18,836 *N*‐glycopeptide MS/MS spectra of Dataset 1. The results demonstrate that the predicted MS/MS spectra for both glycopeptides closely resemble their corresponding experimental MS/MS spectra. DeepGPO can successfully capture the fragmentation pattern of both *N*‐ and *O*‐glycopeptides.

### 2.2 Performance Evaluation for *O*‐Glycopeptide MS/MS Prediction by DeepGPO

**FIGURE 2:**
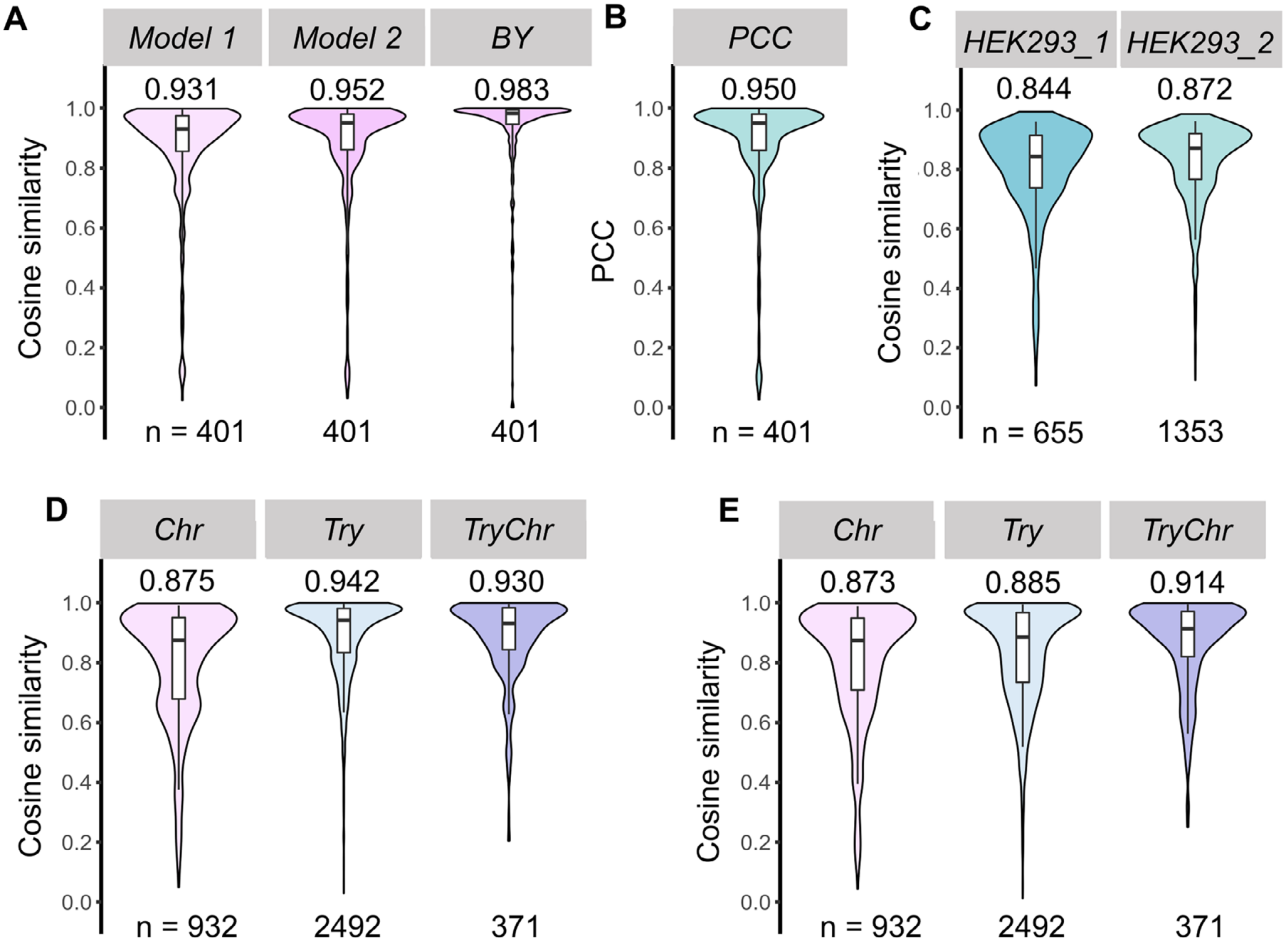
Performance of DeepGPO in MS/MS prediction. (A) The distribution of cosine similarity between predicted and experimental MS/MS spectra of *O*‐glycopeptides from Dataset 1 mouse brain samples. *Model 1*: model without pretraining. *Model 2*: model pretrained on other *O*‐glycoproteomics datasets. *BY*: consideration of only B/Y glycan ions for cosine similarity. (B) The distribution of Pearson correlation coefficient computed between the predicted and experimental MS/MS spectra of *O*‐glycopeptides from Dataset 1 mouse brain samples. The DeepGPO model is the *Model 2* in (A). (C) The distribution of cosine similarity between the predicted and experimental MS/MS spectra of *O*‐glycopeptides from Dataset 1 HEK293 cells. *HEK293_1* represents the parent HCD MS/MS spectra from HCD‐pd‐EThCD and *HEK293_2* represents the HCD MS/MS spectra without triggered EThCD. (D) The distribution of cosine similarity between the predicted and experimental MS/MS spectra for the glycopeptides digested with trypsin (*Try*), chymotrypsin (*Chr*) and the combination of trypsin and chymotrypsin (*TryChr*) from Dataset 3. The DeepGPO was pre‐trained by other *O*‐glycoproteomics datasets, and then fine‐tuned on the data split from Dataset 3. (E) The distribution of cosine similarity between the predicted and experimental MS/MS spectra from Dataset 3. No pre‐training was applied. The medians are indicated. The boxes and whiskers show the quantiles and 95% percentiles, respectively. The numbers of spectra for test are indicated below each graph.

The performance of glycopeptide MS/MS prediction by DeepGPO was first benchmarked using Dataset 1 [29] (Table S1). Dataset 1 employed IMPa which cleaves at the N‐terminal of a serine or threonine residue containing an *O*‐glycan modification. The single‐shot experiments on the mouse brain tissues in Dataset 1 served as the test data, while all the other *O*‐glycopeptides in Dataset 1 served as the training data (Note S1). The glycan types of the test data are shown in Figure S6. Glycopeptides presented in the training data were intentionally removed from the test data to avoid data leakage. We observed a median cosine similarity of 0.931 for *O*‐glycopeptide MS/MS prediction (Figure 2A *Model1*). Moreover, by using the model pre‐trained with other *O*‐glycoproteomics datasets (Dataset 2–11 [10, 11, 30, 31, 32, 33, 34, 35, 36], Table S1) and then fine‐tuned with the training data of Dataset 1, the median cosine similarity can be further improved to 0.952 (Figure 2a *Model2*). We conducted further analysis on the results of Figure 2a *Model2*. Upon narrowing our focus to the glycan B/Y ions, the median cosine similarity increased to 0.983, demonstrating the accurate prediction of B/Y fragment ions intensity (Figure 2a *BY*). Beyond cosine similarity, we further explored the distribution of PCC between the predicted and experimental MS/MS spectra (Figure 2b) with the same setting as Figure 2a *Model2*, and a median PCC of 0.950 was demonstrated.

In another analysis, the *O*‐glycopeptides from HEK293 cells in Dataset 1 were used as the test data, while the *O*‐glycopeptides from the mouse brain samples in Dataset 1 served as the training data. The DeepGPO model was trained first using other *O*‐glycoproteomics datasets (Dataset 2–11 [10, 11, 30, 31, 32, 33, 34, 35, 36, Table S1), and then fine‐tuned on the training data of Dataset 1. For the parent HCD MS/MS spectra from HCD‐pd‐EThCD and the HCD MS/MS spectra (without triggered EThCD) (Note S1), the cosine similarity reached 0.844 and 0.872, respectively (Figure 2c). Given that the organism of the training data was different from the test data, DeepGPO still achieved a cosine similarity of more than 0.84 between predicted and experimental spectra, supporting that DeepGPO trained on non‐human data can generalize to human samples.

In addition to Dataset 1, we also benchmarked DeepGPO on Dataset 3 [31] (PXD004590, Table S1, Note S1), which involved human samples digested with trypsin (*Try*), chymotrypsin (*Chr*) and the combination of trypsin and chymotrypsin (*TryChr*). Each condition served as the test data in turn, with the remaining for training. DeepGPO was first trained with other *O*‐glycoproteomics datasets (Dataset 1–2, 4–11 [10, 11, 29, 30, 32, 33, 34, 35, 36], Table S1) before being fine‐tuned on the data split from Dataset 3. DeepGPO demonstrated remarkable accuracy in predicting *O*‐glycopeptide MS/MS spectra (Figure 2d). When comparing results without pre‐training (Figure 2e), the performance of *O*‐glycopeptides MS/MS prediction on the *Try* data showed the most significant improvement, while that on the *Chr* data remained consistent. This is attributed to the absence of chymotrypsin‐digested peptides in the pre‐training data from Dataset 1–2, 4–11, demonstrating again the significance of pre‐training. For Dataset 3, no *O*‐glycoprotease was used, wherein the *O*‐glycosylation is not necessary on the termini of the peptides.

### 2.3 *O*‐glycosite Localization Using HCD MS/MS Spectra

**FIGURE 3:**
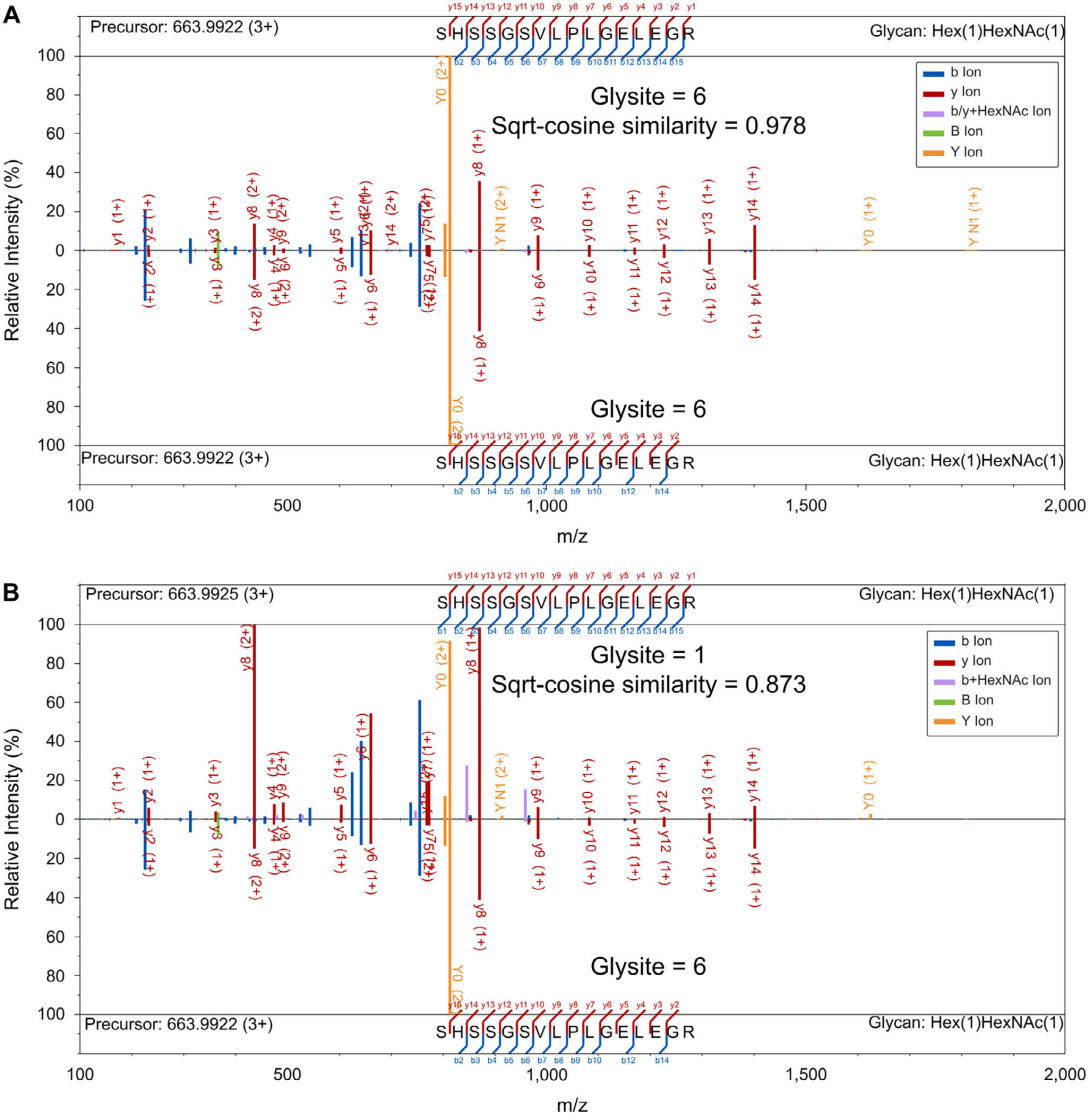
Comparison of experimental and predicted HCD MS/MS spectra of glycopeptides with the same peptide sequence and glycan modification but different glycosites. (A) Glycosite = 6 for both predicted and experimental MS/MS spectra; (B) Glycosite = 1 for predicted MS/MS spectrum and Glycosite = 6 for experimental MS/MS spectrum. Top: the predicted MS/MS spectra; Bottom: the experimental MS/MS spectra.

With the accurate prediction of *O*‐glycopeptides MS/MS spectra, DeepGPO can be used for *O*‐glycosite localization. The principle was first demonstrated through representative examples. Two HCD MS/MS spectra from Dataset 2 [30] (PXD018560, Table S1, Note S1) sharing the same peptide sequence and glycan modification but different glycosites (site 1 or 6) were collected. The sites were verified by the corresponding ETD MS/MS spectra (Figure S7). In silico HCD MS/MS spectra were generated for the two glycopeptides by DeepGPO. As shown in Figure 3 and Figure S8, when the glycosylation sites were consistent between the predicted HCD MS/MS spectrum and the experimental one, a higher spectrum similarity was observed, i.e. sqrt‐cosine similarity of 0.978 for both sites as 6 and 0.975 for both sites as 1; while the similarity was lower when the two sites were different, i.e. 0.873 for predicted site at 1 against experimental site at 6 and 0.849 for predicted site at 6 against experimental site at 1. Further examples are shown in Figure S9.

**FIGURE 4:**
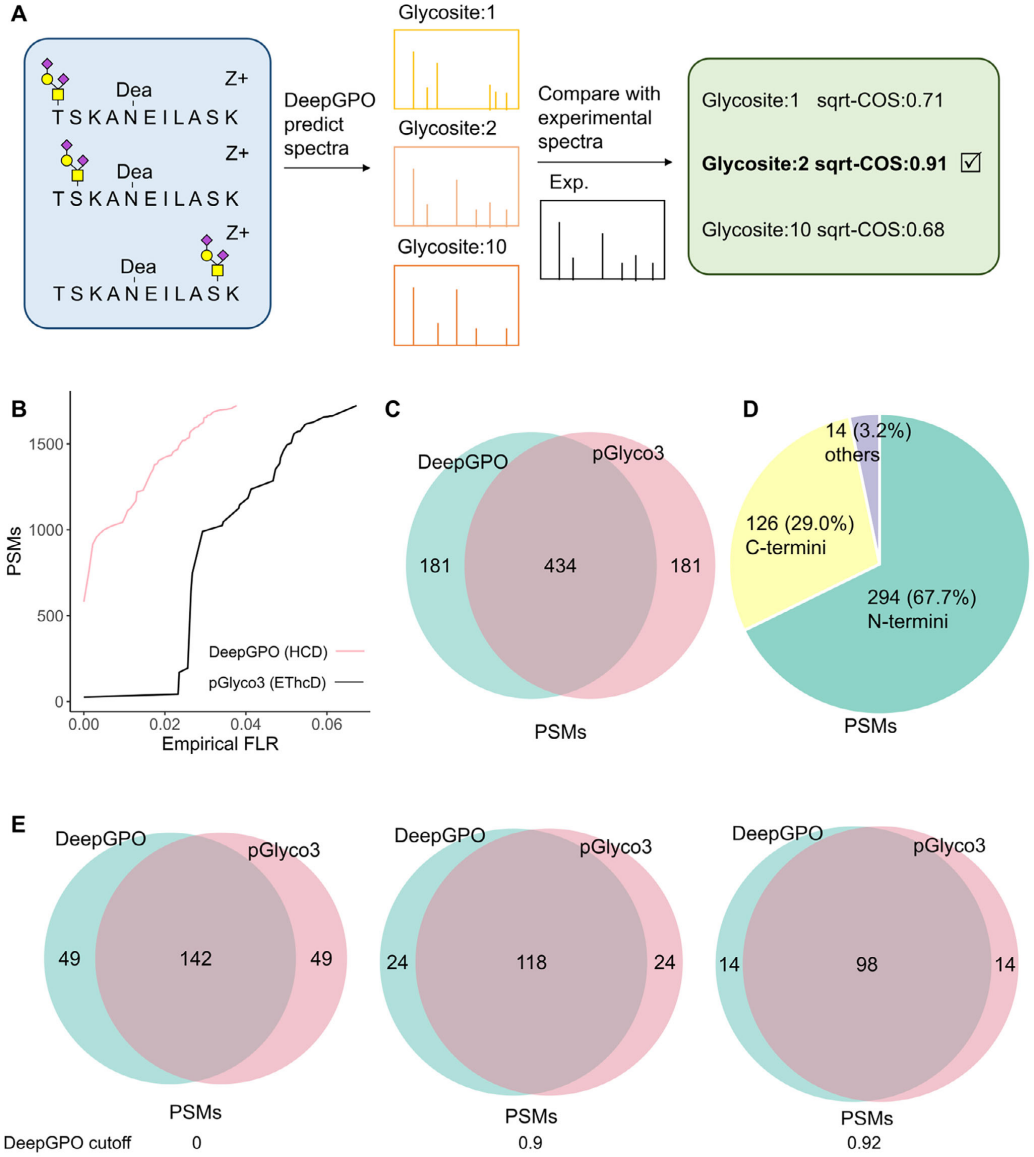
Performance of DeepGPO on glycosite localization. (A) Protocol of performing glycosite localization by DeepGPO. The evaluation was strictly restricted to MS/MS spectra corresponding to glycopeptides with at least two candidate O‐glycosylation sites. (B) Comparison of the number of identified PSMs under a given empirical FLR by DeepGPO from HCD and by pGlyco3 from HCD‐pd‐EThCD for Dataset 1. (C) Venn diagram of the number of PSMs identified by DeepGPO from HCD and by pGlyco3 from HCD‐pd‐EThCD for Dataset 4. (D) Pie chart showing the distribution of PSMs shared by DeepGPO and pGlyco3 in Dataset 4, categorized by N‐termini, C‐termini, and other glycosylation sites. (E) Venn diagram of the number of PSMs identified by DeepGPO with a similarity score cutoff of 0, 0.9 and 0.92, and by pGlyco3 with glycosite localization probability ≥ 0.8 for Dataset 3.

Then, DeepGPO was applied to *O*‐glycosite localization on large‐scale datasets. We first evaluated the possibility using the data of single‐shot experiments on the mouse brain tissues in Dataset 1. The test was performed on the HCD MS/MS spectra of mono‐*O*‐glycosylated peptides with at least two candidate glycosites and starting with Ser/Thr (Figure S10). The candidate glycopeptides list was generated considering all possible glycosites, while the glycan composition assigned by the original database search remains unchanged. MS/MS spectra of all the candidate glycopeptides were generated by DeepGPO. The sqrt‐cosine similarity was calculated between the predicted and experimental MS/MS. The glycopeptide with the highest sqrt‐cosine similarity was reported for each experimental MS/MS (Figure 4a). Due to the use of IMPa *O*‐glycoprotease in sample preparation, the correct glycosites were regarded as N‐termini. Using the parent HCD MS/MS spectra of HCD‐pd‐EThCD, DeepGPO localized 65 out of 1723 tested MS/MS spectra with glycosites different from N‐termini, demonstrating an empirical false localization rate (FLR) of 3.8% (65/1723). In contrast, pGlyco3, using EThCD, localized 116 MS/MS spectra with glycosites different from N‐termini, showing an empirical FLR of 6.7% (116/1723). At a 5% empirical FLR, DeepGPO retained all the 1723 MS/MS spectra, while pGlyco3 identified 1495 MS/MS spectra (Figure 4b, Data S1).

In addition to Dataset 1, DeepGPO was further benchmarked on Dataset 4, where digestions were performed with AM0627 or AM0627 mutants (AM0627^W149A^, AM0627^F290A^, AM0627^Y287A^) against recombinant glycoproteins podocalyxin, MUC16, PSGL‐1, and CD43 [35]. The glycopeptides were analyzed by HCD‐pd‐EThCD. The test data were from the glycopeptides digested with AM0627, which cleaved between adjacent residues carrying truncated core 1 *O*‐glycans. As a result, it is hypothesized that the glycosite was located at either the N‐ or C‐termini of the glycopeptides. From the test data of Dataset 4 (Note S1), we obtained 615 MS/MS spectra of mono‐*O*‐glycosylated peptides with multiple candidate glycosites (Figure S10). DeepGPO, relying solely on HCD MS/MS spectra, localized 131 spectra with glycosites different from either the N or C‐termini of the peptides. In comparison, pGlyco3, using EThCD, localized 125 MS/MS spectra with glycosites different from either the N or C‐termini of the peptides. The Venn diagram of the identification results by pGlyco3 and DeepGPO is shown in Figure 4c. Among the 615 MS/MS spectra, 434 yielded identical results between pGlyco3 and DeepGPO, including 294 (294/434 = 67.7%) with the glycosite at the N‐termini, 126 (126/434 = 29.0%) with the glycosite at the C‐termini, and only 14 (14/434 = 3.2%) with other glycosites (Figure 4d). The overlap results between DeepGPO based on HCD and pGlyco3 based on EThCD are consistent with the specificity of the enzyme. It is noted that although specific enzymatic cleavage should occur between adjacent residues carrying truncated core 1 *O*‐glycans, there can be non‐specific cleavages during enzymatic reaction.

In Dataset 1 and 4, specific *O*‐glycoproteases were used, where the glycosites were located on either N‐ or C‐termini. To demonstrate a more generalized application, we further benchmarked the method on Dataset 3, which consists of EThCD MS/MS spectra of glycopeptides generated with different proteases, including trypsin, chymotrypsin and the combination of trypsin and chymotrypsin, but without *O*‐glycoprotease. The data with the combination of trypsin and chymotrypsin were used as the test data; while the others in the dataset were used as the training data. Glycosylation sites were identified by pGlyco3 using EThCD with localization probability ≥ 0.8. In total, 191 HCD MS/MS spectra of mono‐*O*‐glycosylated peptides with multiple candidate glycosites were collected (Figure S10). The identification results by DeepGPO from HCD MS/MS spectra were largely consistent (142/191 = 74.3%) with the identification results by pGlyco3 with EThCD (Figure 4e). Taken the combined identification results (n = 240) by DeepGPO and pGlyco3, we checked manually the presence of the site‐determining c‐ and z‐type ions in the corresponding EThCD MS/MS spectra. 190 identification results can be found with the diagnostic ions. Among the 190, 150 were identified by DeepGPO using HCD and 168 were identified by pGlyco3 using EThCD (Data S2).

It is noted that the top‐match based on spectra similarity is not always correct. To further improve the identification accuracy by DeepGPO, we applied a cut‐off on the spectra similarity score (sqrt‐cosine similarity). The score distributions for both correct and incorrect peptide‐spectrum‐matches (PSMs) across the three datasets (Datasets 1, 3, and 4) are shown in Figure S11. For Dataset 1, the correct PSMs are considered as those identified with glycosylation on the N‐termini. For Dataset 4, the correct PSMs are considered as those identified with glycosylation on the N‐ or the C‐termini. For Dataset 3, the correct PSMs are considered as those consistent with the pGlyco3 identification results using EThCD (with glycosite localization probability ≥ 0.8). As shown in Figure S11, the distributions of correct and incorrect PSMs are separated at the sqrt‐cosine similarity 0.90 to 0.92. Based on this, we applied the similarity score thresholds of 0.90 and 0.92 to the Dataset 3 identification result by DeepGPO, and further compared to the pGlyco3 identification result (Figure 4e). At the threshold of 0.9, the consistency between DeepGPO and pGlyco3 was 83.1% (118/142). At the threshold of 0.92, the consistency between DeepGPO and pGlyco3 was 87.5% (98/112). Considering that the pGlyco3 identification result can also contain false identification, such consistency between DeepGPO and pGlyco3 is reasonable.

### 2.4 Analysis of Doubly *O*‐glycosylated Peptides by DeepGPO

*O*‐glycoproteomics features challenges in multiple‐glycosylation due to the complexity of assigning glycan modifications to multiple glycosites. Here, we extended the application of DeepGPO to doubly *O*‐glycosylated peptides with the following workflow. First, each glycan is independently embedded using GNN1 to obtain its representation. Second, the representations of the two glycans, together with the peptide sequence and other properties, are fed into the model to generate a global representation of the glycopeptide. Third, when predicting the B/Y ions of a given glycan, GNN2 is used to jointly model the glycan together with the global glycopeptide representation, and to predict the corresponding B/Y ions. In this process, because the glycopeptide representation received by the GNN2 contains information from both glycans, the prediction of the B/Y ions for one glycan is influenced by the other. In the output, DeepGPO predicts more types of fragment ions for the di‐glycosylated peptides than the mono‐glycosylated peptides due to the enlarged combination of various neutral losses (see details in Materials and Methods). It is noted that since many neutral loss ions are not frequently observed, the predicted fragment ions intensity matrix can be sparse. A sparsity stress test as shown in Figure S12 demonstrates that higher sparsity leads to slower early convergence and weaker initial performance, but the final model performance remains comparable. Nevertheless, as part of the future work, the fragment ions for peptides with three or more glycans can be refined based on empirical fragmentation statistics, where ion types that are rarely observed in experiments can be removed to reduce sparsity.

**FIGURE 5:**
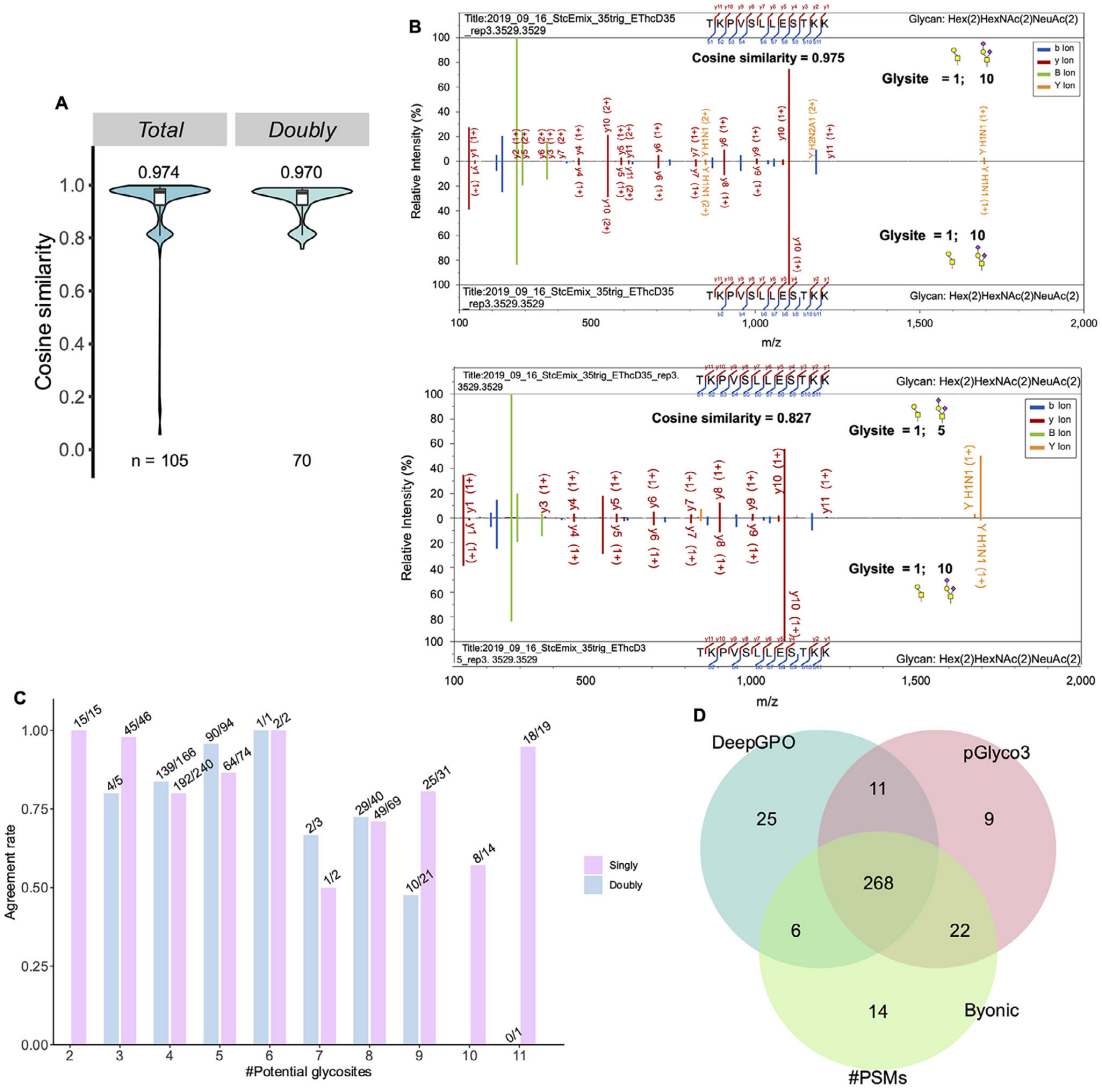
Performance of DeepGPO on multiply *O*‐glycosylated peptides. (A) Cosine similarity between predicted and experimental HCD MS/MS spectra for *O‐*glycopeptides from Dataset 12. *Total*: mono‐ and doubly *O*‐glycopeptides from the test data. *Doubly*: doubly *O‐*glycosylated peptides from the test data. The medians are indicated. The boxes and whiskers show the quantiles and 95% percentiles, respectively. The numbers of spectra for test are indicated below each graph. (B) Example of a doubly *O*‐glycosylated peptide with alternative site localizations. Top: predicted spectrum for glycosylation at positions 1;10 and 1;5; bottom: experimental spectrum corresponding to glycosylation at positions 1;10. (C) Localization consistency of DeepGPO compared to Byonic across peptides with different numbers of serine/threonine residues in Dataset 12. Bars show consistent spectra counts over total, separated by singly (pink) and doubly (blue) glycosylated peptides. (D) Venn diagram showing the overlap of PSMs identified by pGlyco3, Byonic, and DeepGPO in Dataset 12.

Benchmarking of DeepGPO for di‐glycosylated peptides was first performed utilizing a publicly available dataset (Dataset 12, PXD017646, Table S1, Note S1) [37]. HCD‐pd‐ETD and HCD‐pd‐EThCD MS/MS spectra from the dataset for glycopeptides containing no more than two glycosylation modifications were selected for analysis. The MS/MS spectra were partitioned by unique glycopeptides at a 9:1 ratio, resulting in 871 spectra for training and 105 for test. DeepGPO was first trained using other *O*‐glycoproteomics datasets (Dataset 1–11) before being fine‐tuned on the training data of Dataset 12. Identification results reported in the original publication by Byonic were used to label the HCD MS/MS spectra. On the entire test set, DeepGPO achieved a median cosine similarity of 0.974, demonstrating high alignment in HCD MS/MS prediction (Figure 5a). Notably, for the subset of 70 doubly glycosylated peptides in the test set, the model maintained a high median cosine similarity of 0.970, indicating that the performance of HCD MS/MS prediction remained robust even for multiply glycosylated peptides. Compared to Figure 2 of mono‐glycosylated peptides, the similarity distribution in Figure 5a exhibited an additional minor peak at a lower similarity (∼0.8), which can be a result of the limited size of the test dataset. Data sparsity can increase the likelihood of heterogeneous prediction performance, resulting in a secondary peak. It is noted that the identification of di‐glycosylated peptides is more challenging than mono‐glycosylated peptides and the original identification results from search engines can contain misidentifications, especially isomers, which could also influence the deep learning model performance. In general, the model performance for di‐glycosylated peptides is still limited by the availability of high‐quality datasets.

Figure 5b shows examples of the predicted MS/MS spectra of di‐glycosylated peptides with alternative glycosylation sites in comparison to the experimental one. From the example, it is clear that the predicted MS/MS spectrum with correct glycosylation sites presents higher similarity than the incorrect one, similar to the observation in mono‐glycosylated peptides, indicating that spectral similarity based on DeepGPO MS/MS spectra prediction can also be used to assist the localization of glycosylation for di‐glycosylated peptides. We then assessed the localization accuracy of doubly *O‐*glycosylated peptides using DeepGPO. For both singly and doubly *O*‐glycosylated peptides, we enumerated all possible site‐localization patterns across serine and threonine residues, while the glycan composition assigned by the original database search remains unchanged. MS/MS spectra identified as glycopeptides with unambiguous glycosites were excluded. As shown in Figure 5c, DeepGPO achieved high consistency with the original assignments for both singly and doubly glycosylated peptides. To further strengthen the result, we also analyzed Dataset 12 using pGlyco3. A total of 310 MS/MS spectra were identified by both pGlyco3 and Byonic with identical peptide sequences, charge states, and glycans (Figure 5d). Among the 310, 290 were consistently assigned by pGlyco3 and Byonic with the same glycosylation sites. DeepGPO achieved the same localization in 268 of the 290 cases, corresponding to a 92.4% agreement rate, highlighting the model's competitive performance relative to established tools. Notably, among the 127 doubly *O‐*glycosylated peptide MS/MS spectra within the 290 cases, DeepGPO's results matched those of both pGlyco3 and Byonic for 117 spectra, yielding an agreement rate of 92.1% (117/127). It is emphasized that both pGlyco3 and Byonic employed ETD MS/MS spectra for glycosite localization, while DeepGPO used only HCD MS/MS spectra.

### 2.5 Validation and Application of DeepGPO on Clinical Human *O‐*glycoproteomic Samples

**FIGURE 6:**
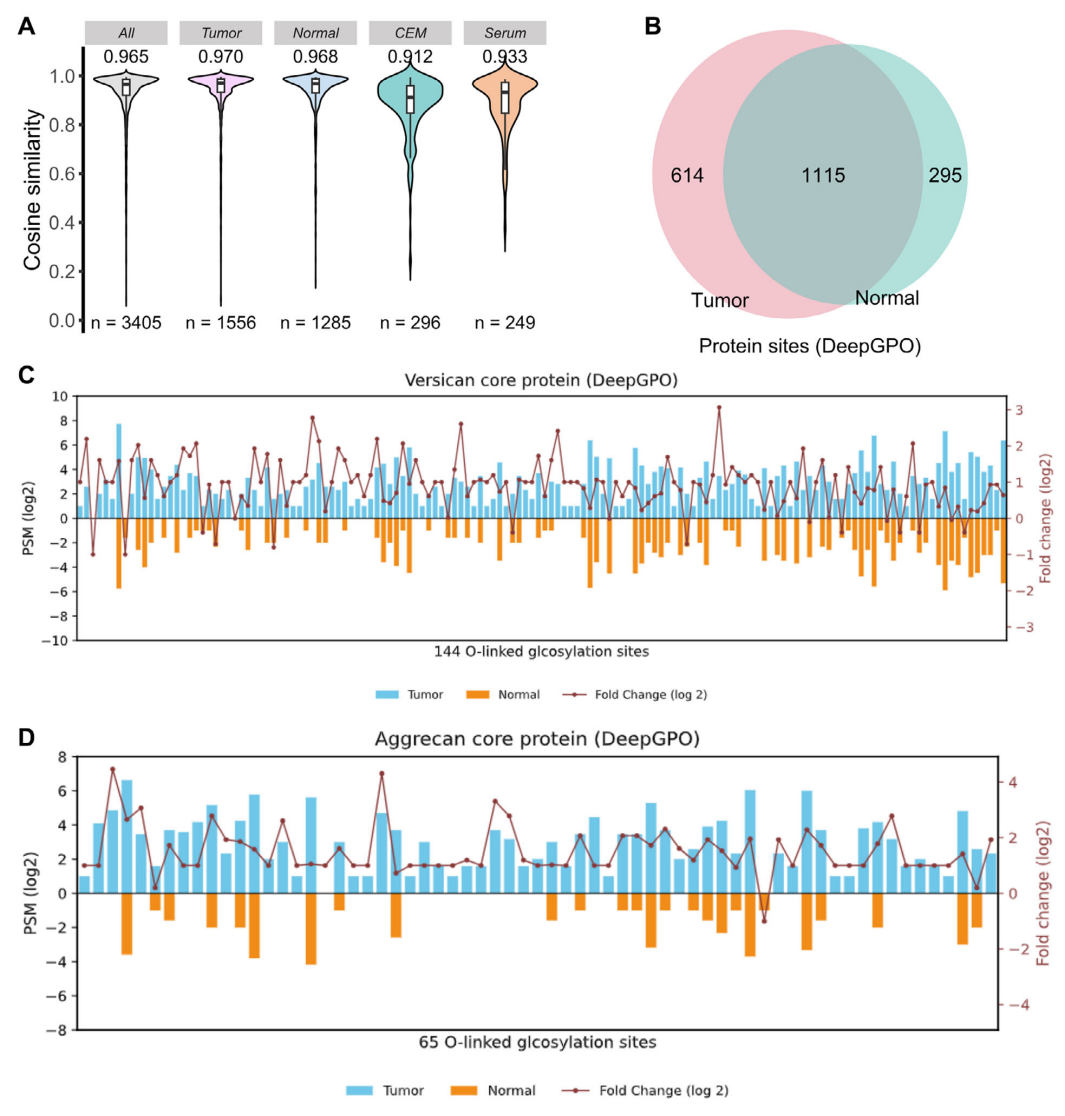
Validation and application of DeepGPO in clinical human *O‐*glycoproteomic samples. (A) Spectral prediction performance on the independent validation set of Dataset 6. Violin plots show the cosine similarity between predicted and experimental *O‐*glycopeptide MS/MS spectra, grouped by sample source. Median cosine similarity values are indicated above each plot. Boxes and whiskers represent quantiles and the 95% percentiles, respectively. The number of spectra in each group is shown below the corresponding plot. (B) The Venn diagram shows shared and sample‐specific *O‐*glycosylation sites between the kidney tumor and adjacent normal tissues after DeepGPO‐assisted site localization. Site‐level comparison of *O‐*glycosylation patterns on (C) versican core protein (VCAN) and (D) aggrecan core protein (ACAN) in kidney tumor and adjacent normal tissues based on DeepGPO‐assisted site assignment.

To further evaluate the performance of DeepGPO on clinically relevant human samples, an *O*‐glycoproteomic dataset, Dataset 6 [11] (PXD009476, Table S1, Note S1) was analyzed. This dataset included paired tumor and adjacent normal kidney tissue samples from patients with clear cell renal cell carcinoma (ccRCC), together with human serum samples and CEM CD4+ T‐cell samples. During model training, Dataset 6 was split at the glycopeptide level at a 9:1 ratio, ensuring no glycopeptide overlap between the training and validation sets. This yielded 34,685 training MS/MS spectra and 3,405 validation MS/MS spectra. DeepGPO was first trained with other *O‐*glycoproteomics datasets (Dataset 1–5, 7–11), and then fine‐tuned on the training set of Dataset 6. On the validation set, DeepGPO achieved a median cosine similarity of 0.965 between predicted and experimental spectra. The validation set included 1,556 spectra from kidney tumor samples, 1,285 spectra from adjacent normal kidney tissue samples, 296 spectra from CEM T‐cell, and 249 spectra from human serum. Across human samples from different biological sources, the median cosine similarity was consistently above 0.9 (Figure 6A), demonstrating the robust generalization ability of DeepGPO across clinically relevant human samples.

DeepGPO was then applied to assist *O*‐glycosylation site localization in kidney tumor and adjacent normal tissue samples. A threshold of sqrt‐cosine similarity 0.92 was used to define confidently refined *O*‐glycosylation sites. The DeepGPO‐refined results revealed tumor and adjacent normal tissue specific *O*‐glycosylation sites (Figure 6B), consistent with the overall distribution observed from the original pGlyco3‐based assignments (Figure S13), while yielding more identified sites. Sequence motif analysis was then performed for the DeepGPO‐refined *O*‐glycosylation sites as well as the original pGlyco3 identification (Figure S14). Consistent with the sequence features reported in the original study of the dataset [11], proline residues were enriched around the modified serine or threonine residues. Site‐level *O*‐glycosylation changes were further examined on versican core protein (VCAN) and aggrecan core protein (ACAN). These two proteins are proteoglycans that normally carry long glycosaminoglycan chains and are highlighted as tumor‐associated *O*‐glycoproteins with extensive *O*‐glycosylation changes [11]. The examination revealed extensive site‐resolved *O*‐glycosylation changes on VCAN and ACAN between tumor and adjacent normal tissue samples based on DeepGPO (Figure 6C,D) and pGlyco3 (Figure S15). DeepGPO and pGlyco3 show similar trends that most of the *O‐*glycosylation sites were up‐regulated in the tumor samples compared to the adjacent normal samples, consistent with the original publication of the dataset [11]. Together, these results demonstrate that DeepGPO achieves high MS/MS spectral prediction accuracy in clinical human *O‐*glycoproteomics samples and facilitates *O*‐glycosylation site localization in human tissues, supporting the applicability of DeepGPO to human disease‐related *O‐*glycoproteomics.

## 3 Discussion

In this work, we present DeepGPO, a deep‐learning model capable of accurately predicting MS/MS spectra for *O*‐glycopeptides. Based on our previous DeepGP model for *N*‐glycopeptides, DeepGPO adopts a number of advanced training techniques to mitigate the limited availability of *O*‐glycopeptides training data. The introduction of a loss re‐weighting method preserves a wider range of MS/MS spectra for model training while assigning greater contributions to more reliable MS/MS spectra during training. Retaining replicate MS/MS spectra provides naturally occurring variation that functions as a form of data augmentation, where the intrinsic variations in different MS/MS spectra for the same glycopeptide are captured by the deep‐learning model. Since many fragmentation events in *N*‐glycopeptides can also happen in *O*‐glycopeptides, DeepGPO also largely benefits from the pre‐trained parameters in DeepGP. Various test datasets were used in this study, including those with and without specific *O*‐glycoprotease digestion, as well as data derived from both human and mouse samples. Even under strict exclusion of glycopeptides available in the training data from the test data, high spectral similarity was still obtained between the experimental and predicted MS/MS spectra. It is noted that the current work focuses on HCD MS/MS spectra prediction, as this fragmentation method is highly efficient and has been adopted by many studies.

With the successful prediction of *O*‐glycopeptides HCD MS/MS spectra, we assessed the ability to localize *O*‐glycosylation by spectra matching. DeepGPO challenges the prevailing assumption that HCD fragmentation alone is insufficient for the localization of *O*‐glycosites [29, 38]. Unlike conventional sequence database searching, which relies on the observation of site‐determining ions to identify *O*‐glycosites, DeepGPO captures both m/z and intensity variations, offering superior performance in the analysis of *O*‐glycopeptides. Three different datasets were used to demonstrate the principle, including one with IMPa *O*‐glycoprotease digestion to generate *O*‐glycopeptides starting with Ser/Thr and glycosylation at the N‐termini, one with mucin‐selective metalloprotease AM0627 which cleaves between adjacent residues carrying truncated core 1 *O*‐glycans, and one without any specific *O*‐glycoprotease. DeepGPO yielded favorable results of *O*‐glycosite localization based solely on HCD spectra. When the similarity score threshold was set to 0.90 or 0.92, higher consistency was observed between the DeepGPO results based on HCD and the pGlyco3 results based on EThCD. The results demonstrate that DeepGPO together with HCD can effectively identify *O*‐glycosites, largely consistent with the enzyme specificity and the ETD‐based site‐localization. Since HCD fragmentation can achieve greater coverage of glycopeptides due to faster cycle time and more efficient dissociation [29], this advancement has the potential to enhance *O*‐glycoproteomics.

The primary limitation of DeepGPO arises from the limited availability of high‐quality datasets for model training and validation. Synthetic peptides are generally considered gold standards for studying fragmentation patterns, but comprehensive datasets for synthetic *O*‐glycopeptides remain scarce. This limits several downstream applications, including the estimation of false discovery rates (FDRs) at the glycopeptide level. In our application of *O*‐glycosites localization, the lack of synthetic glycopeptides datasets also hampers further validation. Besides, the current work has primarily focused on singly glycosylated peptides, with preliminary investigations extended to di‐glycosylated peptides. At present, DeepGPO cannot effectively process peptides with three or more glycan attachments, owing to limited training data and increased spectral complexity. In addition, although DeepGPO is not explicitly constrained to a specific glycan type at the model design level, its current implementation and evaluation are based on mucin‐type *O*‐glycosylation also due to the availability of data. With expanded datasets and continued advances in analytical tools, DeepGPO has the potential for broader application in glycoproteomics research.

In summary, DeepGPO is developed for the prediction of *O*‐glycopeptides MS/MS spectra and has shown potential in localizing *O*‐glycosites. With the development of glycoproteomics, particularly in mass spectrometry methods and data analysis software solutions, we anticipate that DeepGPO will enhance the understanding of the heterogeneity and complexity of glycoproteins, and further aid biologists in the study of glycobiology.

## 4 Materials and Methods

### 4.1 Glycoproteome Data Analysis by pGlyco3

Glycoproteomic data analysis was performed using pGlyco3 (version: pGlyco3.1, https://github.com/pFindStudio/). For *O*‐glycoproteomics data analysis, HCD‐pd‐EThCD or EThCD MS/MS spectra were analyzed using the pGlyco‐*O‐*Glycan.gdb glycan database, while for HCD spectra, the protein sequence FASTA file, glycan database, and modification settings were consistent with those described in the original publications of the corresponding datasets. For *N*‐glycoproteomics data analysis, the glycan type was specified as *N*‐glycan, and the glycan database was pGlyco‐*N‐*Human.gdb. Analytical parameters were set as follows: precursor mass tolerance at 5 ppm, fragment mass tolerance at 20 ppm, and glycopeptide FDR at 0.01. All the other parameters were kept at their default settings.

### 4.2 DeepGPO Framework

The DeepGPO framework is based on DeepGP [24]. The performance of MS/MS spectra prediction is evaluated using sqrt‐cosine similarity:

$$\mathrm{Sqrt}\;\mathrm{cosine}\;\mathrm{similatity}=\frac{\sum_{k=1}^{l_{ij}}\sqrt{y_{ik}y_{jk}}}{\sqrt{\sum_{k=1}^{n_{i}}y_{ik}\times \sum_{k=1}^{n_{j}}y_{jk}}}$$

where *l_ij_* represents the number of common peaks between spectrum *i* and spectrum *j*, while *n_i_* and *n_j_* denote the counts of peaks identified as glycopeptide fragments in spectra *i* and *j*, respectively. The variable *y* indicates peak intensity. A tolerance of 20 ppm is applied to identify common peaks, and in cases where multiple peaks fall within this tolerance, the peak closest in distance is selected.

DeepGPO predicts 24 types of peptide b/y fragment peaks, denoted as b1, b1n, b1o, b1h, b1nh, b1oh, y1, y1n, y1o, y1h, y1nh, y1oh, b2, b2n, b2o, b2h, b2nh, b2oh, y2, y2n, y2o, y2h, y2nh, y2oh. The first character indicates the ion type (b or y ions), followed by the fragment charge (1 or 2). The last character(s) specify the type of neutral loss: “o” represents the loss of H_2_O, “n” indicates loss of NH_3_, and “h” denotes loss of a monosaccharide HexNAc. In our analysis, peaks without the “h” symbol correspond to b/y fragments containing one HexNAc moiety, while those with the “h” symbol represent b/y fragments that have lost the HexNAc moiety. This nomenclature is designed to ensure that each symbol clearly indicates the type of loss, simplifying coding. DeepGPO recognizes 16 types of glycan B/Y fragment peaks (B1, B1n, B1o, B1f, Y1, Y1n, Y1o, Y1f, B2, B2n, B2o, B2f, Y2, Y2n, Y2o, Y2f). The first character indicates the ion type (B or Y ions), and “f” signifies the loss of a monosaccharide Fuc, with the other symbols adhering to the same nomenclature rules of b/y fragment peaks. The total number of peaks is determined by the number of cleavage events and the types of ions associated with each event. Notably, ions that are theoretically impossible are not excluded from model predictions; instead, predicting these ions results in penalties within our scoring system, which encourages the model to refine its learning. However, during the post‐processing phase, ions identified as theoretically impossible are omitted when converting the output matrix into the corresponding predicted MS/MS spectra. Importantly, there is no restriction on precursor charge states, but we consider fragment charges up to +2.

### 4.3 DeepGPO Model Training

DeepGPO uses the search results by pGlyco3 for model training, validation and test except where otherwise specified. Glycopeptides present in the training data are excluded from the validation and test data. The weight of the MS/MS spectra used for model training should exceed 0.5. During model training, the MS/MS spectra intensity is normalized by the highest peak in the spectrum. The model utilizes a modified Mean Squared Error (MSE) loss function. Specifically, for spectra with assigned weights, the loss function is multiplied by the corresponding weight. Training of DeepGPO on Dataset 1 took 1 h using a single RTX 3090 GPU with 100 epochs, a batch size of 256, and a learning rate of 1 × 10^−4^. DeepGPO took 1 s to predict 300 spectra using a single RTX 3090 GPU.

### 4.4 DeepGPO Evaluation

The experimental MS/MS spectra are converted to Mascot generic format (.mgf). DeepGPO generates the predicted MS/MS spectra. A tolerance of 20 ppm is established to identify common peaks between the experimental and predicted MS/MS spectra. When multiple peaks fall within this range, the peak with the shortest distance is selected. Any discrepancies between the compared spectra result in the missing peak intensity being assigned a value of 0, followed by the calculation of spectrum similarity. DeepGPO provides various metrics for this assessment, including square root cosine similarity (sqrt‐cosine), cosine similarity (COS), and Pearson correlation coefficient (PCC).

### 4.5 Analysis of Doubly Glycosylated Peptides

For the analysis of doubly glycosylated peptides, the identification results were obtained from the original study using Byonic. The same filtering criteria as in the original study were applied to ensure consistency and comparability. Specifically, peptide‐spectrum matches were retained if they met the following conditions: a Byonic score ≥ 200, a logProb value ≥ 2, and a peptide length of more than four amino acid residues.

The model framework of DeepGPO for doubly glycosylated peptides is consistent with that used for singly glycosylated peptides. To handle two glycans, each glycan is independently embedded using one GNN (GNN1). When predicting the B/Y ions of a given glycan, a new GNN (GNN2) is used to jointly model the glycan together with the global glycopeptide representation, and to predict the corresponding B/Y ions. DeepGPO predicts 36 types of b/y fragment ions for di‐glycosylated peptides, including: b1, b1n, b1o, b1h, b1nh, b1oh, b1hh, b1nhh, b1ohh, y1, y1n, y1o, y1h, y1nh, y1oh, y1hh, y1nhh, y1ohh, b2, b2n, b2o, b2h, b2nh, b2oh, b2hh, b2nhh, b2ohh, y2, y2n, y2o, y2h, y2nh, y2oh, y2hh, y2nhh, y2ohh. The interpretation of the characters remains the same as in the singly glycosylated peptide model.

### 4.6 Implementation and Visualization

DeepGPO was developed using Python (3.8.3, Anaconda distribution version 5.3.1, https://www.anaconda.com/) with the following packages: bidict (0.22.0), dgl (2.4.0+cu118), FastNLP (0.6.0), numpy (1.18.5), pandas (1.0.5), pyteomics (4.7.3), pytorch (1.8.1), torchinfo (1.7.1), transformers (4.12.5), scikit‐learn (1.5.2) and scipy (1.14.1). Visualization was performed using custom scripts in R (4.0.2) with the following packages: VennDiagram (1.6.20) and ggplot2 (3.3.2). The mirrored spectra were plotted by GP‐plotter [39] (1.0.0).

### 4.7 Motif Analysis and Site‐Level Comparison in the Analysis of the Clinical Glycoproteomic Data

For the clinical glycoproteomic data analysis, *O*‐glycosylation sequence motifs were analyzed using pLogo [40]. Fifteen‐amino‐acid sequence windows centered on the modified serine or threonine residues were extracted from the identified *O*‐glycosylation sites. Sites without sufficient flanking sequences were excluded from the analysis. Amino acid enrichment around the modification sites was visualized using pLogo, with human protein sequences used as the background.

Site‐level *O*‐glycosylation changes between the kidney tumor and adjacent normal tissue samples were analyzed using spectral counting. Spectra assigned to VCAN and ACAN were extracted separately from tumor and adjacent normal tissue samples, and site‐level spectral counts were summarized according to the assigned *O*‐glycosylation sites. For visualization, spectral counts were log2‐transformed to improve the display of low‐abundance sites. For fold‐change calculation, spectral counts were normalized by the total number of PSMs in each individual sample. Tumor‐to‐normal fold change was calculated as the normalized tumor spectral count divided by the normalized normal spectral count and expressed as log2 fold‐change. Sites detected exclusively in tumor or normal samples were assigned fixed log2 fold‐change values of 1 or −1. Low‐confidence sites with a maximum spectral count of no more than one across all comparison groups (DeepGPO tumor, DeepGPO normal, pGlyco3 tumor, pGlyco3 normal) were excluded.

### 4.8 Data and Code Availability

All data are available in the main text or the supplementary materials. The raw datasets used in this study are available in the ProteomeXchange Consortium (https://www.proteomexchange.org/) under accession code PXD037415 [29], PXD018560 [30], PXD004590 [31], PXD032164 [35], PXD022896 [32], PXD009476 [11], PXD020077 [10], PXD039583 [33], PXD027616 [34], PXD031225 [35], PXD035775 [36] and PXD017646 [37].

DeepGPO along with the user guide is freely available via https://gitfront.io/r/yuz2011/e7B9HHrM8V52/DeepGPO/ and GitHub [https://github.com/lmsac/DeepGPO]. A compiled standalone version of the software is provided, along with a demonstration video and testing models, at the following link: https://drive.google.com/drive/folders/1frGwRgUSPbBMk27uYDrf4wdqn6KSzEjb.

## Author Contributions

Y.Z., Y.W., and L.Q. conceived the study. Y.Z. and Y.W. implemented the code. Y.Z. performed the data analysis and wrote the first draft of the manuscript. L.Q. reviewed and edited the manuscript and supervised all aspects of the work.

## Conflicts of Interest

The authors declare no conflicts of interest.

## Supporting information




**Supporting File 1**: advs76760‐sup‐0001‐SuppMat.pdf.

**Supporting File 2**: advs76760‐sup‐0002‐Data S1.xlsx.

**Supporting File 3**: advs76760‐sup‐0003‐Data S2.xlsx.

## Data Availability

The data that support the findings of this study are available in ProteomeXchange at https://www.proteomexchange.org/. These data were derived from the following resources available in the public domain: PXD037415, https://proteomecentral.proteomexchange.org/ui?pxid=PXD037415; PXD018560, https://proteomecentral.proteomexchange.org/ui?pxid=PXD018560; PXD004590, https://proteomecentral.proteomexchange.org/ui?pxid=PXD004590; PXD032164, https://proteomecentral.proteomexchange.org/ui?pxid=PXD032164; PXD022896, https://proteomecentral.proteomexchange.org/ui?pxid=PXD022896; PXD009476, https://proteomecentral.proteomexchange.org/ui?pxid=PXD009476; PXD020077, https://proteomecentral.proteomexchange.org/ui?pxid=PXD020077; PXD039583, https://proteomecentral.proteomexchange.org/ui?pxid=PXD039583; PXD027616, https://proteomecentral.proteomexchange.org/ui?pxid=PXD027616; PXD031225, https://proteomecentral.proteomexchange.org/ui?pxid=PXD031225; PXD035775, https://proteomecentral.proteomexchange.org/ui?pxid=PXD035775; PXD017646, https://proteomecentral.proteomexchange.org/ui?pxid=PXD017646.

## References

[1] M. Ma , R. Dubey , A. Jen , et al., “Regulated N‐Glycosylation Controls Chaperone Function and Receptor Trafficking,” Science 386, no. 6722, (2024): 667–672, 10.1126/science.adp7201. 39509507 PMC7617332

[2] I. S. B. Larsen , Y. Narimatsu , H. J. Joshi , et al., “Discovery of an O‐Mannosylation Pathway Selectively Serving Cadherins and Protocadherins,” Proceedings of the National Academy of Sciences 114, no. 42, (2017): 11163–11168, 10.1073/pnas.1708319114. PMC5651762 28973932

[3] T. M. Block , M. A. Comunale , M. Lowman , et al., “Use of Targeted Glycoproteomics to Identify Serum Glycoproteins That Correlate With Liver Cancer in Woodchucks and Humans,” Proceedings of the National Academy of Sciences 102, no. 3, (2005): 779–784, 10.1073/pnas.0408928102. PMC545516 15642945

[4] S. Yang , J. Li , J. Zhang , et al., “Recent Advances in the Analysis of Protein Glycosylation by Hydrophilic Interaction Liquid Chromatography‐Mass Spectrometry,” SSRN (2022), 10.2139/ssrn.4001304.

[5] H. Hu , K. Khatri , J. Klein , N. Leymarie , and J. Zaia , “A Review of Methods for Interpretation of Glycopeptide Tandem Mass Spectral Data,” Glycoconjugate Journal 33, no. 3, (2016): 285–296, 10.1007/s10719-015-9633-3. 26612686 PMC4882288

[6] D. Bojar and F. Lisacek , “Glycoinformatics in the Artificial Intelligence Era,” Chemical Reviews 122, no. 20, (2022): 15971–15988, 10.1021/acs.chemrev.2c00110. 35961636 PMC9615983

[7] W. F. Zeng , W. Q. Cao , M. Q. Liu , S. M. He , and P. Y. Yang , “Precise, Fast and Comprehensive Analysis of Intact Glycopeptides and Modified Glycans With pGlyco3,” Nature Methods 18, no. 12, (2021): 1515–1523, 10.1038/s41592-021-01306-0. 34824474 PMC8648562

[8] D. A. Polasky , F. C. Yu , G. C. Teo , and A. I. Nesvizhskii , “Fast and Comprehensive N‐ and O‐glycoproteomics Analysis With MSFragger‐Glyco,” Nature Methods 17, no. 11, (2020): 1125–1132, 10.1038/s41592-020-0967-9. 33020657 PMC7606558

[9] L. Lu , N. M. Riley , M. R. Shortreed , C. R. Bertozzi , and L. M. Smith , “O‐Pair Search With MetaMorpheus for O‐glycopeptide Characterization,” Nature Methods 17, no. 11, (2020): 1133–1138, 10.1038/s41592-020-00985-5. 33106676 PMC7606753

[10] N. M. Riley , S. A. Malaker , and C. R. Bertozzi , “Electron‐Based Dissociation is Needed for O‐Glycopeptides Derived From OpeRATOR Proteolysis,” Analytical Chemistry 92, no. 22, (2020): 14878–14884, 10.1021/acs.analchem.0c02950. 33125225 PMC8329938

[11] W. Yang , M. Ao , Y. Hu , Q. K. Li , and H. Zhang , “Mapping the O‐Glycoproteome Using Site‐Specific Extraction of O‐Linked Glycopeptides (EXoO),” Molecular Systems Biology 14, no. 11, (2018): 8486, 10.15252/msb.20188486. PMC6243375 30459171

[12] S. Vainauskas , H. Guntz , E. McLeod , et al., “A Broad‐Specificity O‐Glycoprotease That Enables Improved Analysis of Glycoproteins and Glycopeptides Containing Intact Complex O‐Glycans,” Analytical Chemistry 94, no. 2, (2022): 1060–1069, 10.1021/acs.analchem.1c04055. 34962767

[13] S. A. Malaker , K. Pedram , M. J. Ferracane , et al., “The Mucin‐Selective Protease StcE Enables Molecular and Functional Analysis of Human Cancer‐Associated Mucins,” Proceedings of the National Academy of Sciences 116, no. 15, (2019): 7278–7287, 10.1073/pnas.1813020116. PMC6462054 30910957

[14] S. Toghi Eshghi , P. Shah , W. Yang , X. Li , and H. Zhang , “GPQuest: A Spectral Library Matching Algorithm for Site‐Specific Assignment of Tandem Mass Spectra to Intact N‐Glycopeptides,” Analytical Chemistry 87, no. 10, (2015): 5181–5188, 10.1021/acs.analchem.5b00024. 25945896 PMC4721644

[15] W. F. Zeng , X. X. Zhou , W. J. Zhou , H. Chi , J. F. Zhan , and S. M. He , “MS/MS Spectrum Prediction for Modified Peptides Using pDeep2 Trained by Transfer Learning,” Analytical Chemistry 91, no. 15, (2019): 9724–9731, 10.1021/acs.analchem.9b01262. 31283184

[16] X. X. Zhou , W. F. Zeng , H. Chi , et al., “pDeep: Predicting MS/MS Spectra of Peptides With Deep Learning,” Analytical Chemistry 89, no. 23, (2017): 12690–12697, 10.1021/acs.analchem.7b02566. 29125736

[17] C. Tarn and W. F. Zeng , “pDeep3: Toward More Accurate Spectrum Prediction With Fast Few‐Shot Learning,” Analytical Chemistry 93, no. 14, (2021): 5815–5822, 10.1021/acs.analchem.0c05427. 33797898

[18] S. Gessulat , T. Schmidt , D. P. Zolg , et al., “Prosit: Proteome‐Wide Prediction of Peptide Tandem Mass Spectra by Deep Learning,” Nature Methods 16, no. 6, (2019): 509–518, 10.1038/s41592-019-0426-7. 31133760

[19] S. Tiwary , R. Levy , P. Gutenbrunner , et al., “High‐Quality MS/MS Spectrum Prediction for Data‐Dependent and Data‐Independent Acquisition Data Analysis,” Nature Methods 16, no. 6, (2019): 519–525, 10.1038/s41592-019-0427-6. 31133761

[20] Y. Yang , X. Liu , C. Shen , Y. Lin , P. Yang , and L. Qiao , “In Silico Spectral Libraries by Deep Learning Facilitate Data‐Independent Acquisition Proteomics,” Nature Communications 11, no. 1, (2020): 146, 10.1038/s41467-019-13866-z. PMC6952453 31919359

[21] R. H. Lou , W. Z. Liu , R. J. Li , S. S. Li , X. M. He , and W. Q. Shui , “DeepPhospho Accelerates DIA Phosphoproteome Profiling Through in Silico Library Generation,” Nature Communications 12, no. 1, (2021): 6685, 10.1038/s41467-021-26979-1. PMC8602247 34795227

[22] Y. Zong , Y. Wang , Y. Yang , et al., “DeepFLR Facilitates False Localization Rate Control in Phosphoproteomics,” Nature Communications 14, no. 1, (2023): 2269, 10.1038/s41467-023-38035-1. PMC10119288 37080984

[23] Y. Yang and Q. Fang , “Prediction of Glycopeptide Fragment Mass Spectra by Deep Learning,” Nature Communications 15, no. 1, (2024): 2448, 10.1038/s41467-024-46771-1. PMC10951270 38503734

[24] Y. Zong , Y. Wang , X. Qiu , X. Huang , and L. Qiao , “Deep Learning Prediction of Glycopeptide Tandem Mass Spectra Powers Glycoproteomics,” Nature Machine Intelligence 6, no. 8, (2024): 950–961, 10.1038/s42256-024-00875-x.

[25] J. Liu , A. W. Bell , J. J. Bergeron , et al., “Methods for Peptide Identification by Spectral Comparison,” Proteome Science 5, no. 1, (2007): 3, 10.1186/1477-5956-5-3. 17227583 PMC1783643

[26] H. Jiang and O. Nachum , “Identifying and Correcting Label Bias in Machine Learning,” Proceedings of Machine Learning Research 108 (2020): 702–712..

[27] A. Vaswani , N. Shazeer , N. Parmar , et al., Advances in Neural Information Processing Systems, (NIPS, 2017).

[28] J. Devlin , M.‐W. Chang , K. Lee , and K. Toutanova , “BERT: Pre‐Training of Deep Bidirectional Transformers for Language Understanding,” in Proceedings of the 2019 Conference of the North American Chapter of the Association for Computational Linguistics: Human Language Technologies, Minneapolis, MN, USA 2019, pp, 4171–4186, 10.18653/v1/N19-1423.

[29] S. Suttapitugsakul , Y. Matsumoto , R. P. Aryal , and R. D. Cummings , “Large‐Scale and Site‐Specific Mapping of the Murine Brain O‐Glycoproteome With IMPa,” Analytical Chemistry 95, no. 36, (2023): 13423–13430, 10.1021/acs.analchem.3c00408. 37624755 PMC10501376

[30] T. D. Madsen , L. H. Hansen , J. Hintze , et al., “An Atlas of O‐linked Glycosylation on Peptide Hormones Reveals Diverse Biological Roles,” Nature Communications 11, no. 1, (2020): 4033, 10.1038/s41467-020-17473-1. PMC7441158 32820167

[31] S. L. King , H. J. Joshi , K. T. Schjoldager , et al., “Characterizing the O‐glycosylation Landscape of human Plasma, Platelets, and Endothelial Cells,” Blood Advances 1, no. 7, (2017): 429–442, 10.1182/bloodadvances.2016002121. 29296958 PMC5738978

[32] Y. Zhang , W. J. Zhao , Y. H. Mao , et al., “O‐Glycosylation Landscapes of SARS‐CoV‐2 Spike Proteins,” Frontiers in Chemistry 9 (2021): 689521, 10.3389/fchem.2021.689521. 34552909 PMC8450404

[33] J. Chongsaritsinsuk , A. D. Steigmeyer , K. E. Mahoney , et al., “Glycoproteomic Landscape and Structural Dynamics of TIM family Immune Checkpoints Enabled by Mucinase SmE,” Nature Communications 14, no. 1, (2023): 6169, 10.1038/s41467-023-41756-y. PMC10550946 37794035

[34] K. Pedram , N. N. Laqtom , D. J. Shon , et al., “Lysosomal Cathepsin D Mediates Endogenous Mucin Glycodomain Catabolism in Mammals,” Proceedings of the National Academy of Sciences 119, no. 39, (2022): 2117105119, 10.1073/pnas.2117105119. PMC9522329 36122205

[35] D. J. Shon , D. Fernandez , N. M. Riley , M. J. Ferracane , and C. R. Bertozzi , “Structure‐Guided Mutagenesis of a Mucin‐Selective Metalloprotease From Akkermansia muciniphila Alters Substrate Preferences,” Journal of Biological Chemistry 298, no. 5, (2022): 101917, 10.1016/j.jbc.2022.101917. 35405095 PMC9118916

[36] N. M. Riley and C. R. Bertozzi , “Deciphering O‐glycoprotease Substrate Preferences With O‐Pair Search,” Molecular Omics 18, no. 10, (2022): 908–922, 10.1039/d2mo00244b. 36373229 PMC10010678

[37] N. M. Riley , S. A. Malaker , M. D. Driessen , and C. R. Bertozzi , “Optimal Dissociation Methods Differ for N‐ and O‐Glycopeptides,” Journal of Proteome Research 19, no. 8, (2020): 3286–3301, 10.1021/acs.jproteome.0c00218. 32500713 PMC7425838

[38] S. A. Malaker , N. M. Riley , D. J. Shon , et al., “Revealing the Human Mucinome,” Nature Communications 13, no. 1, (2022): 3542, 10.1038/s41467-022-31062-4. PMC9209528 35725833

[39] Z. Fang , M. Dong , H. Qin , and M. Ye , “GP‐Plotter: Flexible Spectral Visualization for Proteomics Data With Emphasis on Glycoproteomics Analysis,” Genomics, Proteomics & Bioinformatics 22, no. 5, (2024): qzae069, 10.1093/gpbjnl/qzae069. PMC11661977 39378133

[40] J. P. O'Shea , M. F. Chou , S. A. Quader , J. K. Ryan , G. M. Church , and D. Schwartz , “pLogo: A Probabilistic Approach to Visualizing Sequence Motifs,” Nature Methods 10, no. 12, (2013): 1211–1212, 10.1038/nmeth.2646. 24097270

