# Supplementary material for: Deep Learning Prediction of O‐Glycopeptide Tandem Mass Spectra Enhances O‐Glycoproteomics
Source: Adv Sci (Weinh). 2026 Jul 23;13(58):e76760. doi: 10.1002/advs.76760 (PMC13393516; doi:10.1002/advs.76760)
Supplement: Supplementary file 1 [file ADVS-13-e76760-s002.pdf]

Supporting Information

Deep Learning Prediction of *O*-Glycopeptide Tandem Mass Spectra Enhances *O*-Glycoproteomics

Yu Zong[1,2], *Yuxin Wang*[3], *Liang Qiao*[1,*]

1. Department of Chemistry, and Minhang Hospital, Fudan University, Shanghai, China.

2. Cancer Science Institute of Singapore, National University of Singapore, Singapore, Singapore

3. Department of Computer Science, and Institute of Modern Languages and Linguistics, Fudan University, Shanghai, China.

*: Correspondence should be addressed to Dr. Liang Qiao (liang_qiao@fudan.edu.cn)

Table of Contents

Supplementary Note: Description of the datasets used **S3**

Fig. S1. The model architecture of DeepGPO **S5**

Fig. S2. Workflow for assigning training weights to glycopeptide MS/MS spectra **S6**

Fig. S3. Effect of loss re-weighting on model performance. **S7**

Fig. S4. Performance of DeepGP, BERT and Transformer as the base models for DeepGPO **S8**

Fig. S5. Performance of DeepGP and Finetuned-DeepGP as the base models for DeepGPO **S9**

Fig. S6. The glycan compositions for the glycopeptides identified from Dataset 1 **S10**

Fig. S7. Comparison of the experimental ETD MS/MS of the glycopeptides of the same peptide sequence and glycan composition but different glycosites **S11**

Fig. S8. Comparison of experimental and predicted HCD MS/MS of glycopeptides with the same peptide sequence and glycan modification but different glycosites, example 1 **S12**

Fig. S9. Comparison of experimental and predicted HCD MS/MS of glycopeptides with the same peptide sequence and glycan modification but different glycosites, example 2 **S13**

Fig. S10. The proportion of MS/MS spectra corresponding to glycopeptides with different numbers of candidate *O*-glycosylation sites across three datasets **S14**

Fig. S11. The score distributions for both correct and incorrect PSMs **S15**

Fig. S12. Performance evaluation of sparsity augmentation in DeepGPO training **S16**

Fig. S13. The Venn diagram shows shared and sample-specific *O*-glycosylation sites between kidney tumor and adjacent normal tissues by pGlyco3 **S17**

Fig. S14. Analysis of amino acid sequence surrounding *O*-linked glycosylation sites **S18**

Fig. S15. Site-level comparison of *O*-glycosylation patterns on VCAN and ACAN based on pGlyco3 **S19**

Table S1. Datasets for deep learning model training, evaluation and test **S20**

Reference **S21**

Supplementary Note

DeepGPO was mainly benchmarked on Dataset 1, Dataset 2, Dataset 3, Dataset 4, Dataset 6 and Dataset 12. Dataset 1 is accessed through ProteomeXchange with identifier PXD037415[1]. *O*-glycopeptides from HEK293 cells or mouse brain tissues were released by treating the samples with IMPa *O*-glycoprotease. For the HEK293 cells, HCD-pd-EThCD or HCD alone was used for glycopeptide fragmentation. For the mouse brain tissues, only HCD-pd-EThCD was used for glycopeptide fragmentation. In the dataset, there were data from (a) single-shot experiments, (b) experiments where the peptide load was increased, and (c) experiments with a modified MS1 scan range. The same mouse brain tissue was used for both *N*- and *O*-glycoproteomics analyses including (a) *O*-glycoproteomics analyses using HCD-pd-EthCD, and (b) *N*-glycoproteomics analyses using sceHCD with collision energies of 20%, 30% and 40%.

Dataset 2 is accessed through ProteomeXchange with identifier PXD018560[2]. The original study contained samples from human, rat and pig. In our study, datasets of human and pig were used. ETD triggering of subsequent HCD scan was used for the experiments.

Dataset 3 is accessed through ProteomeXchange with identifier PXD004590[3]. *O*-glycopeptides from human plasma, platelets and endothelial cells were treated with trypsin and/or chymotrypsin. MS/MS analysis was performed using HCD and ETD.

Dataset 4 is accessed through ProteomeXchange with identifier PXD032164[4]. Digestion was performed with AM0627 or AM0627 mutants ($AM0627^{W149A}$, $AM0627^{F290A}$, $AM0627^{Y287A}$) against recombinant glycoproteins, podocalyxin, MUC16, PSGL-1, and CD43. The glycopeptides were analyzed by HCD-pd-EThCD.

Dataset 6 is accessed through ProteomeXchange with identifier PXD009476[5]. This dataset was generated using the site-specific extraction of *O*-linked glycopeptides strategy to map *O*-linked glycosylation sites and their corresponding glycans in human samples. Proteins from human kidney tissues, serum, and CEM T cells were digested. For kidney tissue, paired tumor and adjacent normal tissues from patients with clear cell renal cell carcinoma were analyzed.

Dataset 12 is accessed through ProteomeXchange with identifier PXD017646[6]. In this study, multiple dissociation methods were employed for glycoproteomics analysis. We

analyzed the *O*-glycopeptide data generated using HCD-pd-ETD and HCD-pd-EThcD. The Byonic search results provided in the original publication was used.

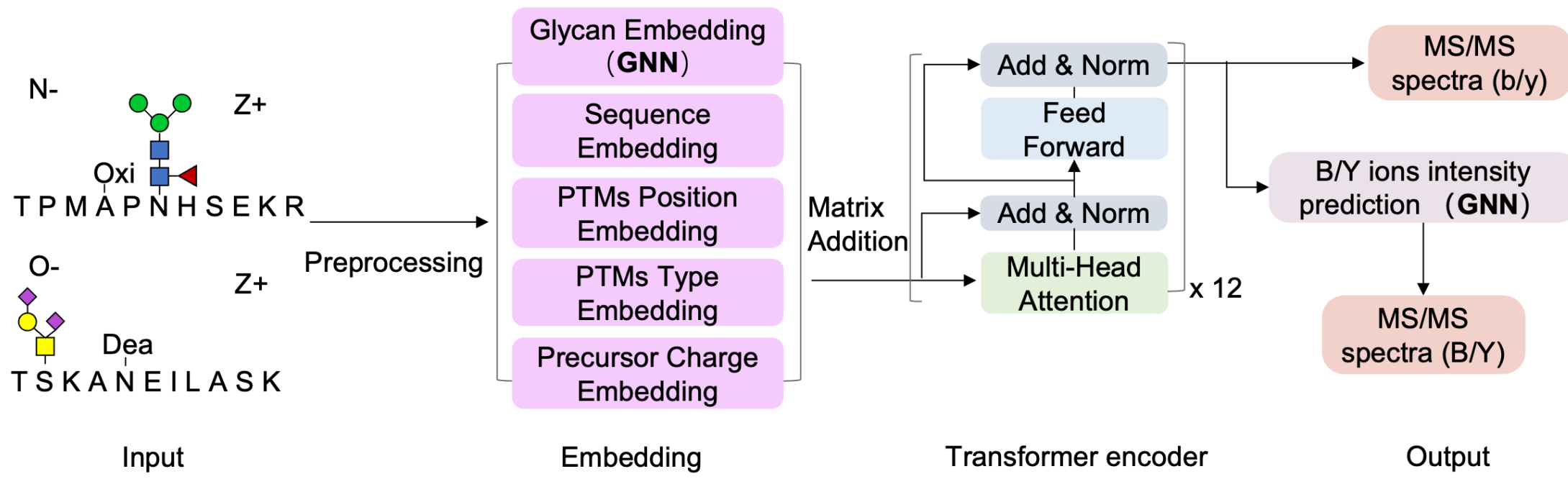

Figure S1. The model architecture of DeepGPO.

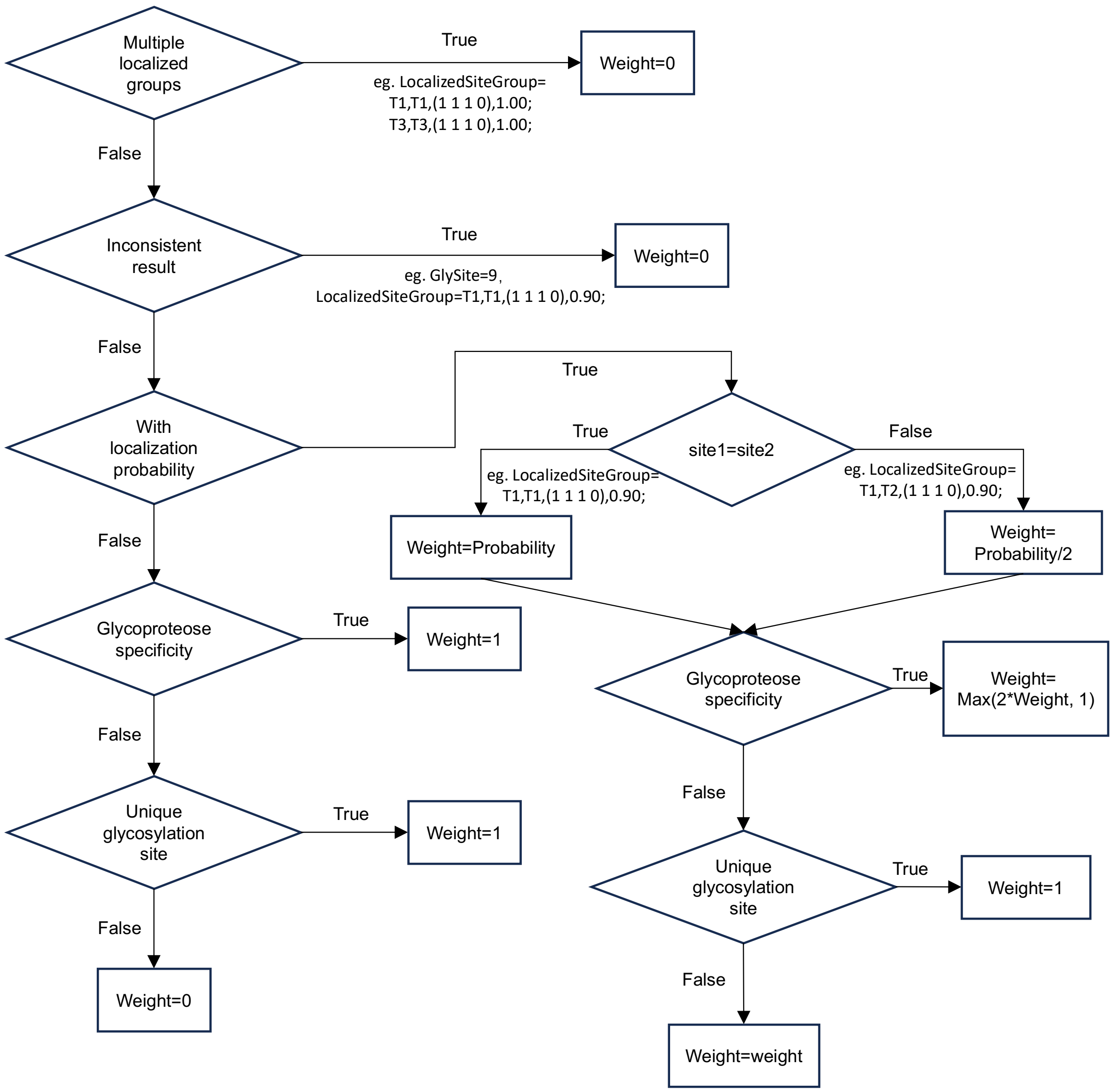

Figure S2. Workflow for assigning training weights to glycopeptide MS/MS spectra. The weights are only applied to the mono-glycosylated peptides. An initial weight is obtained from the pGlyco3 as the localization probability. When the reported glycosite is inconsistent with those in the LocalizedSiteGroup, the weight is 0. Spectra without localization probability due to the lack of ETD data are assigned the weight of 0. If site1 is the same as site2 in the LocalizedSiteGroup, the weight is the probability. If site1 differs from site2, the weight of each site is halved. When specific glycoprotease is used, for pGlyco3 identification results that match glycoprotease specificity, the weight is double of the weight without considering the glycoprotease specificity, ensuring a minimum weight of 1. Glycopeptides with only one potential glycosylation site receive the weight of 1.

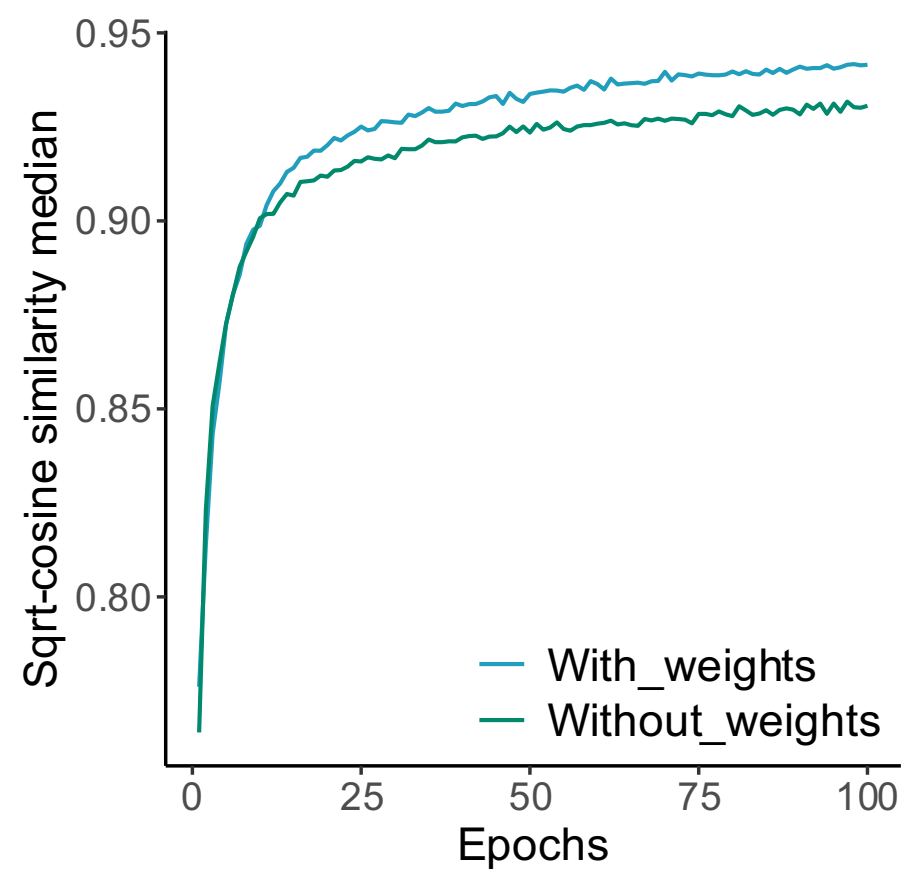

Figure S3. Effect of loss re-weighting on model performance. Comparison of DeepGPO training on Dataset 1 with and without the loss re-weighting strategy. The unweighted setting assigns equal weight (weight = 1) to all MS/MS spectra, whereas the weighted setting uses confidence-based weights derived from glycopeptide identification and enzymatic specificity as detailed in **Figure S2**.

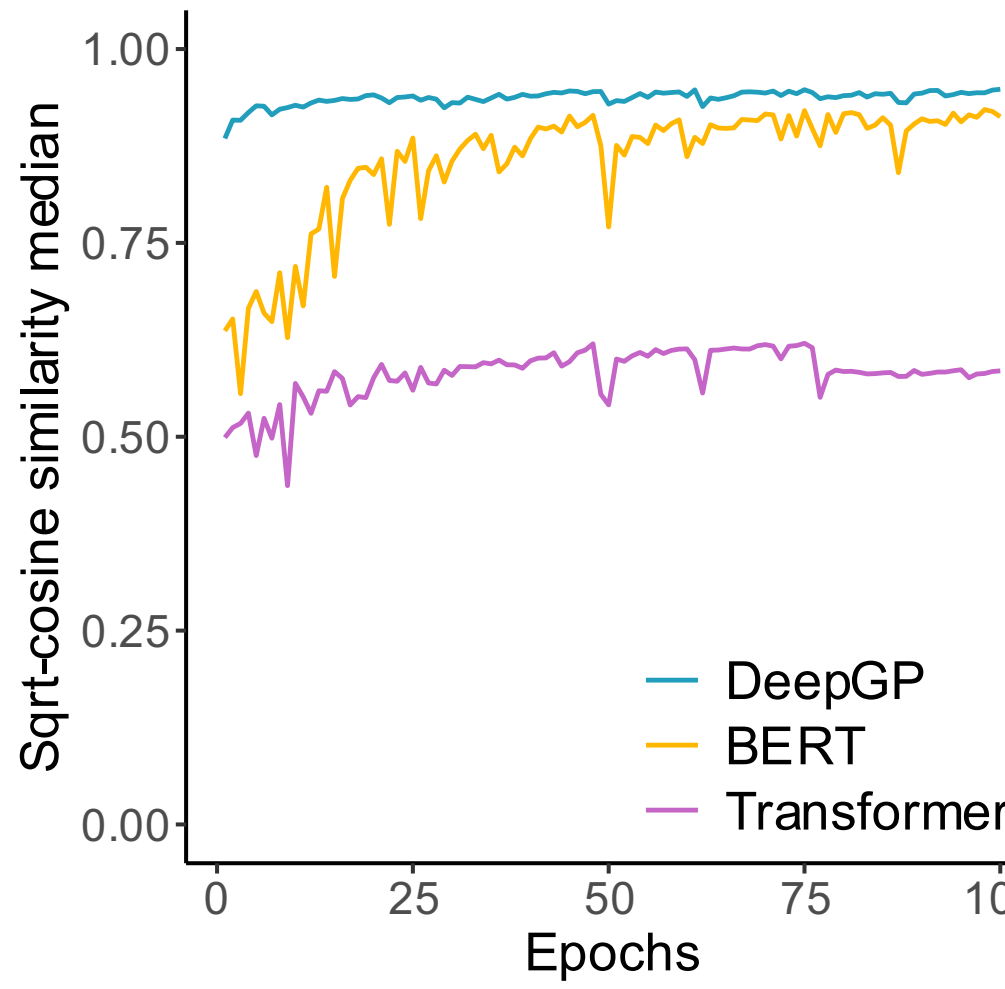

Figure S4. Performance of DeepGP, BERT and Transformer as the base models for DeepGPO training using Dataset 1.

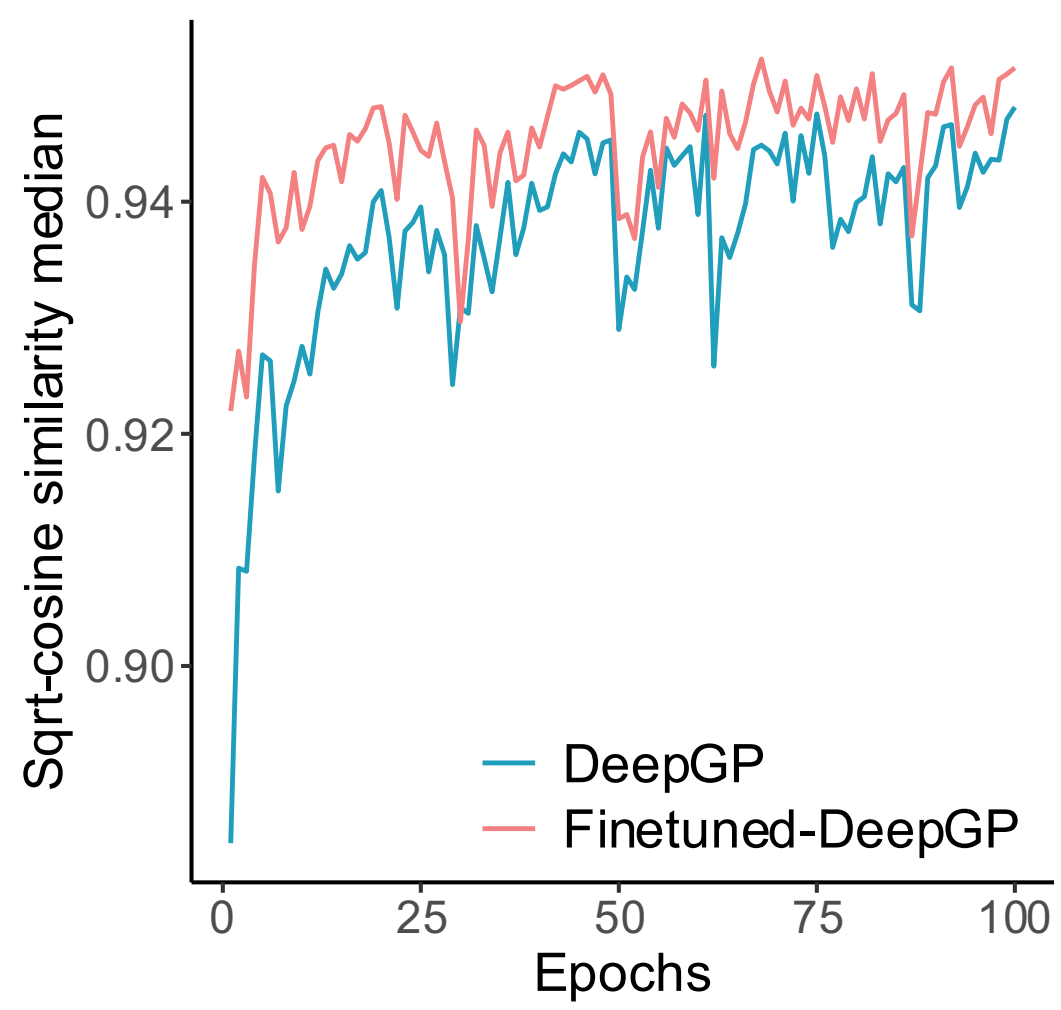

Figure S5. Performance of DeepGP and Finetuned-DeepGP as the base models for DeepGPO training using Dataset 1. Finetuned-DeepGP means that the base model is DeepGP trained with other *O*-glycopeptides datasets (Dataset 2-11).

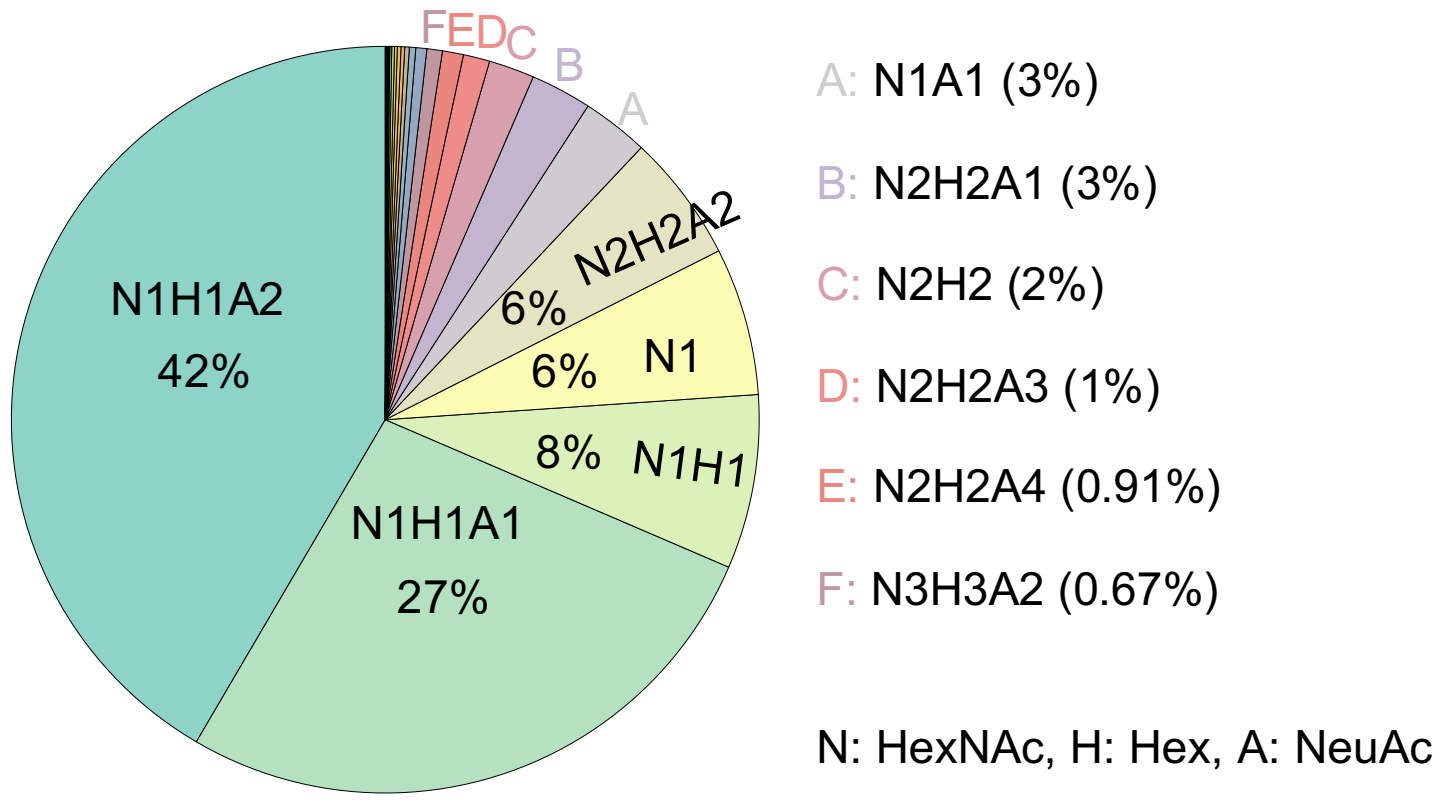

Figure S6. Distribution of glycan compositions for glycopeptides identified from Dataset 1. Compositions with relative abundances >0.5% are annotated. Monosaccharides are denoted in a condensed format: H, hexose (Hex); N, N-acetylhexosamine (HexNAc); A, N-acetylneuraminic acid (NeuAc).

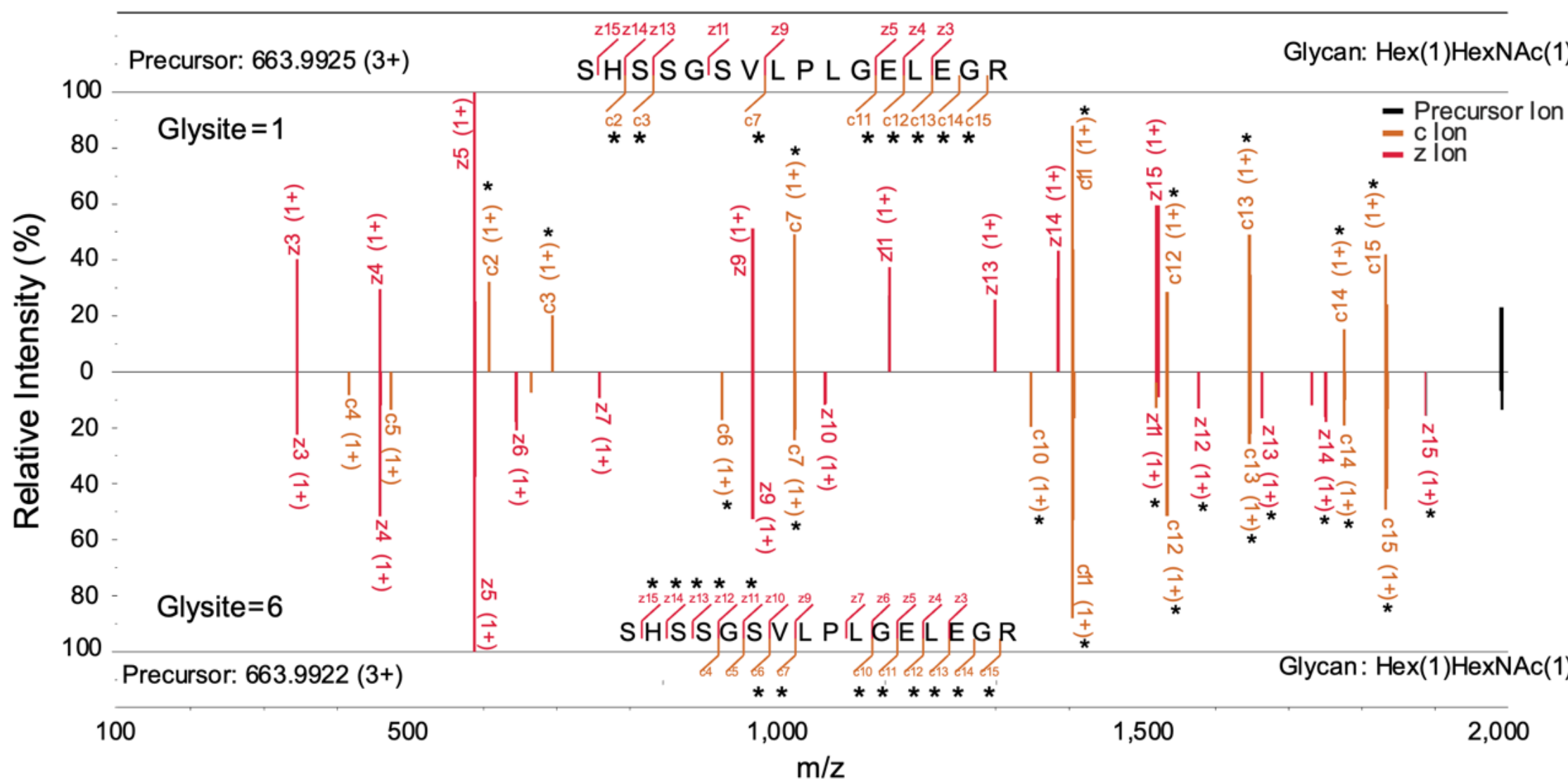

Figure S7. Comparison of the experimental ETD MS/MS of the glycopeptides of the same peptide sequence and glycan composition but different glycosites. Top: the experimental MS/MS spectra with the glycosite at the first position; Bottom: the experimental MS/MS spectra with the glycosite at the sixth position. *: fragments with the intact glycan.

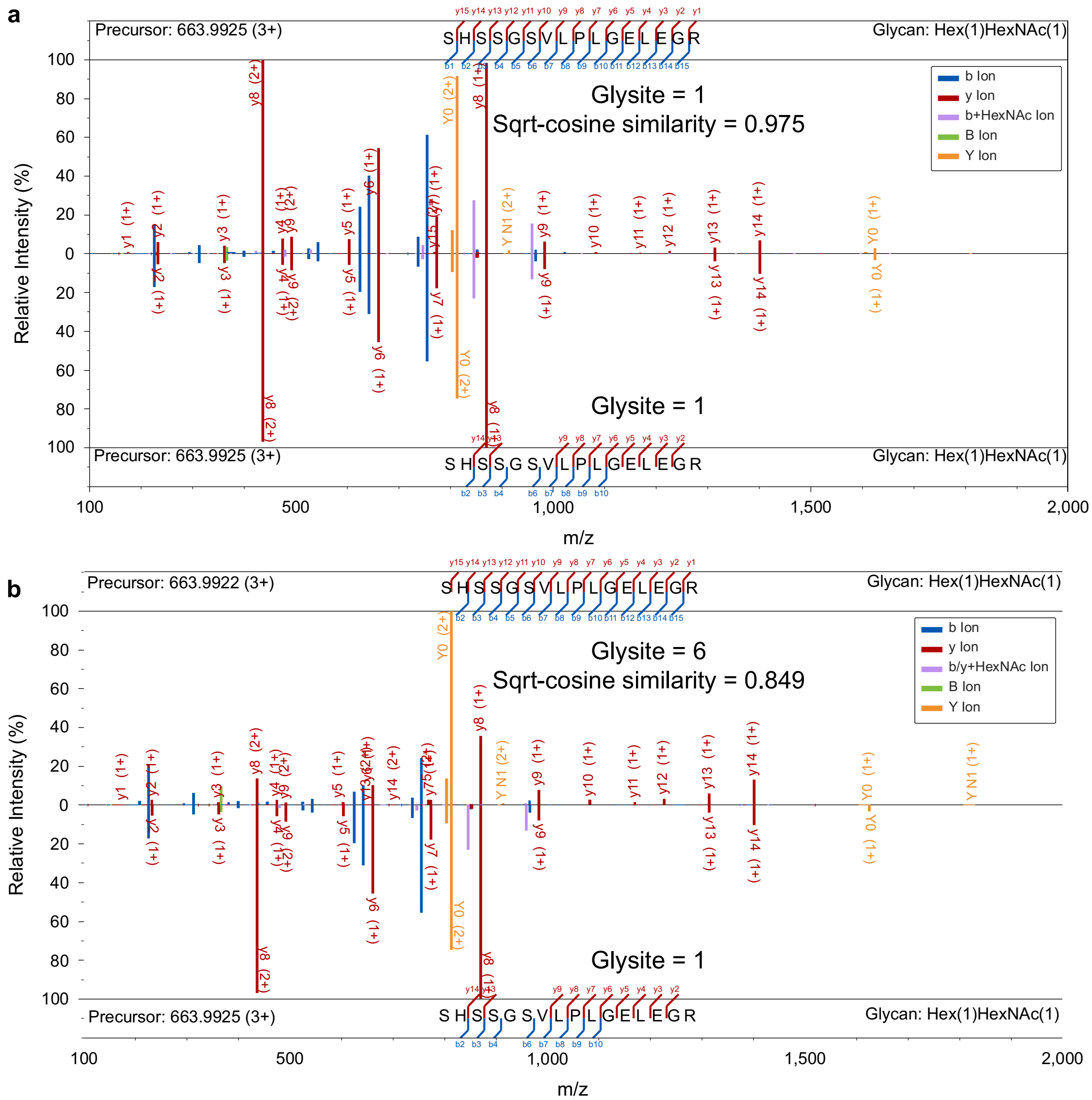

Figure S8. Comparison of experimental and predicted HCD MS/MS of glycopeptides with the same peptide sequence and glycan modification but different glycosites. (a) Glycosite = 1 for both predicted and experimental MS/MS spectra; (b) Glycosite = 6 for predicted MS/MS spectra and Glycosite = 1 for experimental MS/MS spectra. Top: the predicted MS/MS spectra; Bottom: the experimental MS/MS spectra.

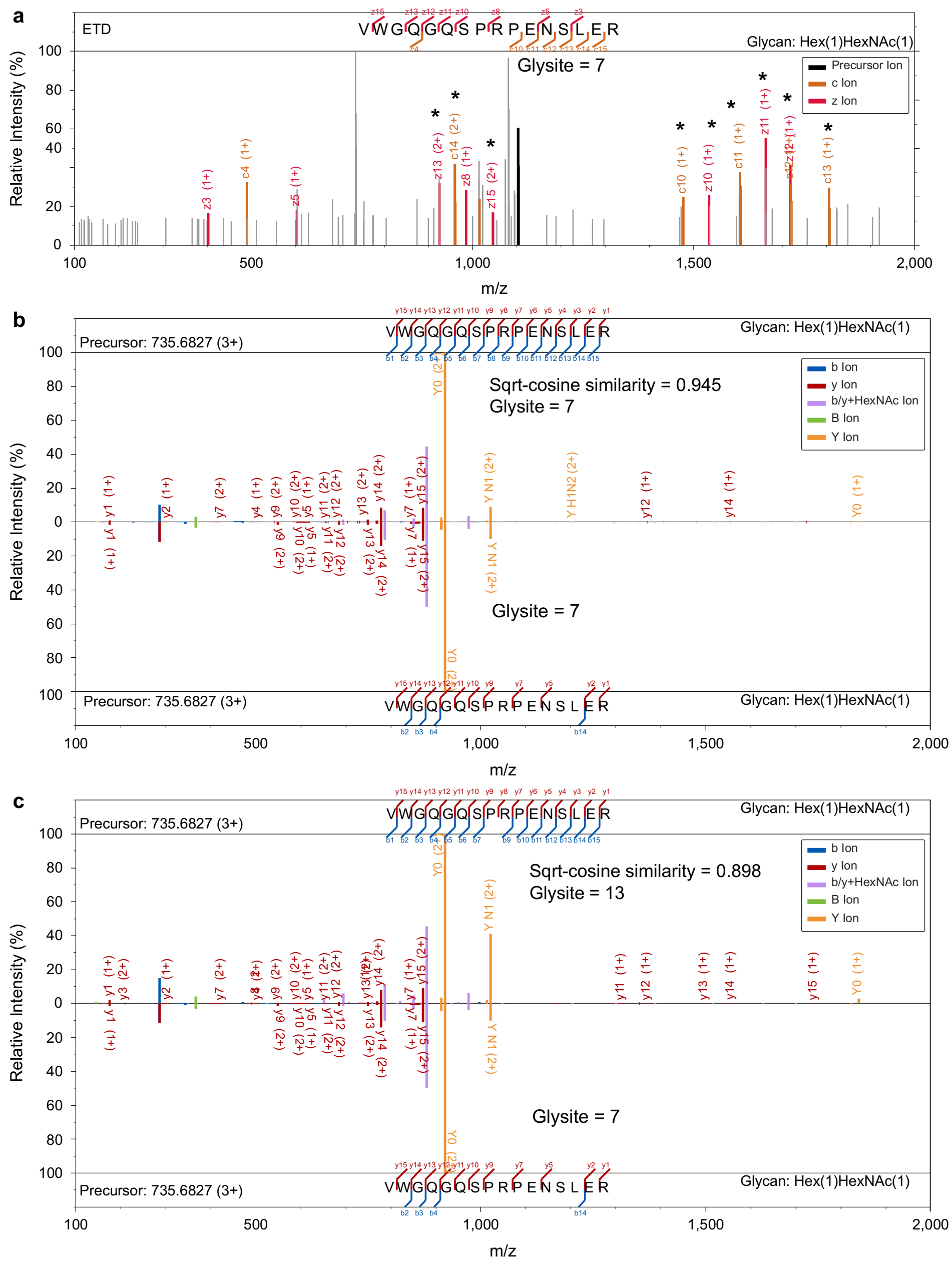

Figure S9. Comparison of experimental and predicted HCD MS/MS of glycopeptides with the same peptide sequence and glycan modification but different glycosites. (a) The ETD MS/MS spectrum of a glycopeptide, demonstrating the glycosylation at site 7. (b) Comparison between the predicted HCD MS/MS spectrum (top) of the glycopeptide with site at 7 to the experimental HCD MS/MS spectrum (bottom) of the glycopeptide. (c) Comparison between the predicted HCD MS/MS spectrum (top) of a glycopeptide with the same peptide sequence and glycan composition but a site at 13 to the experimental HCD MS/MS spectrum (bottom) of the glycopeptide.

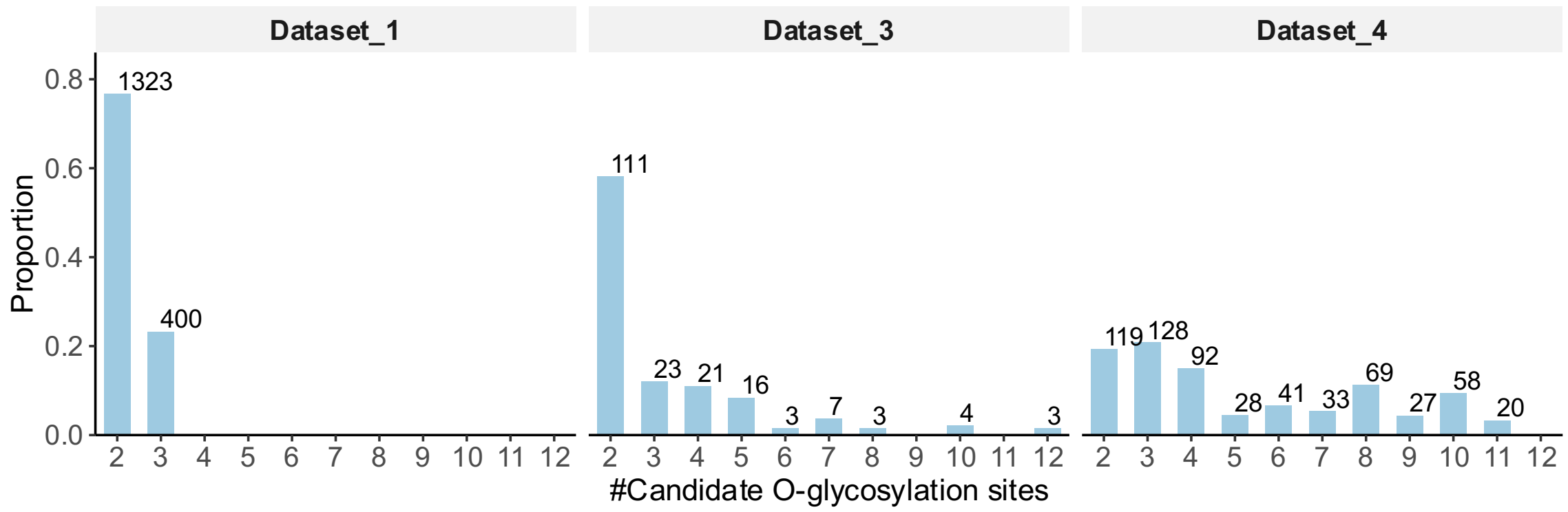

Figure S10. Bar plots showing the proportion of MS/MS spectra corresponding to glycopeptides with different numbers of candidate *O*-glycosylation sites across the test data of three datasets relative to **Figure 4**. Each panel represents one dataset, with bars indicating the relative proportion of spectra assigned to each site count category. Numbers above the bars denote the corresponding spectra counts.

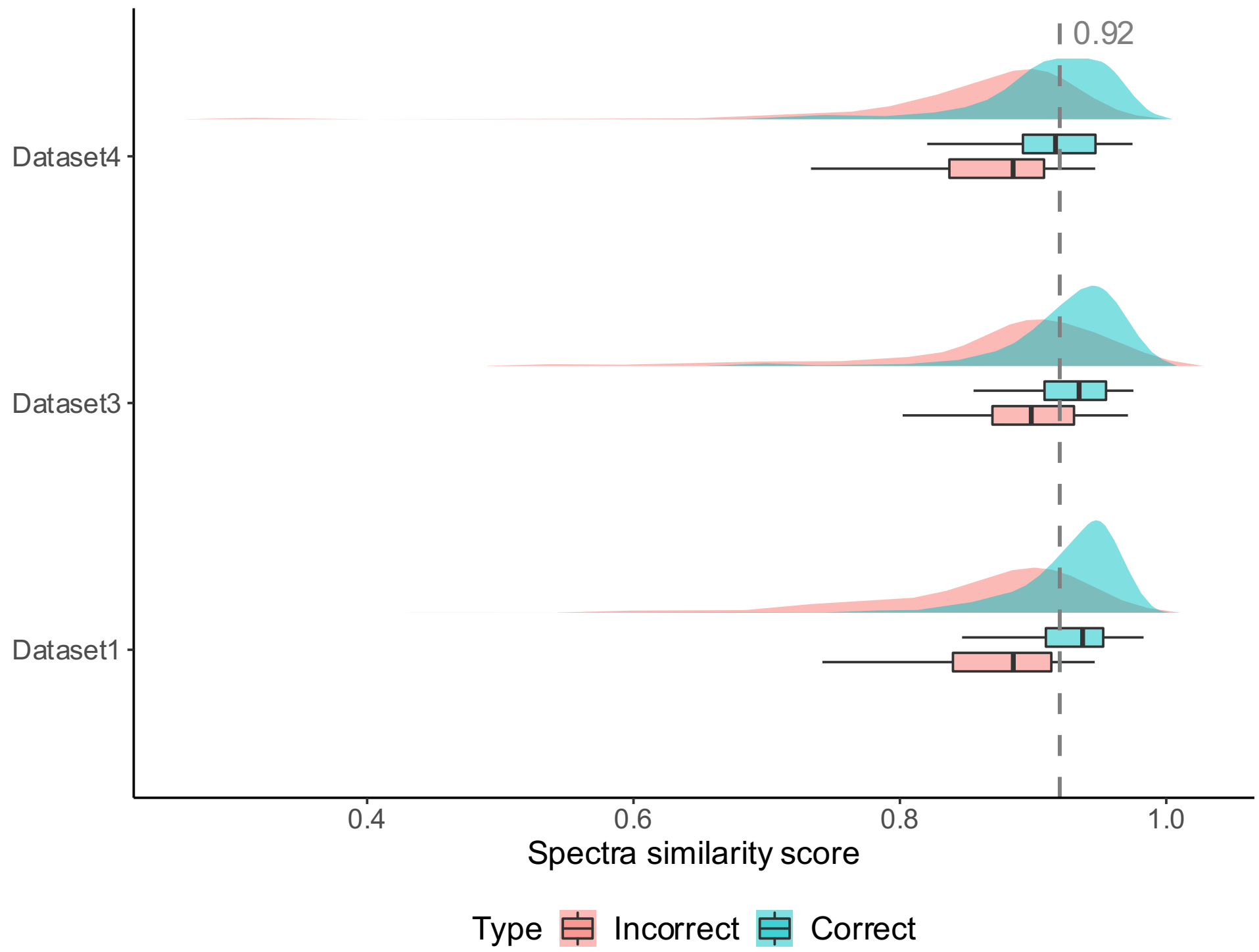

Figure S11. The score distributions (sqrt cosine similarity) for both correct and incorrect PSMs from three datasets (Dataset 1, Dataset 3 and Dataset 4). Half violin graph (Top) and boxplot (Bottom) of sqrt cosine similarity distribution for correct and incorrect glycopeptides hits. Boxes mark the first and third quartile, with the median highlighted as the line, and whiskers mark the minimum/maximum values within the 1.5 interquartile range. Outliers are not shown.

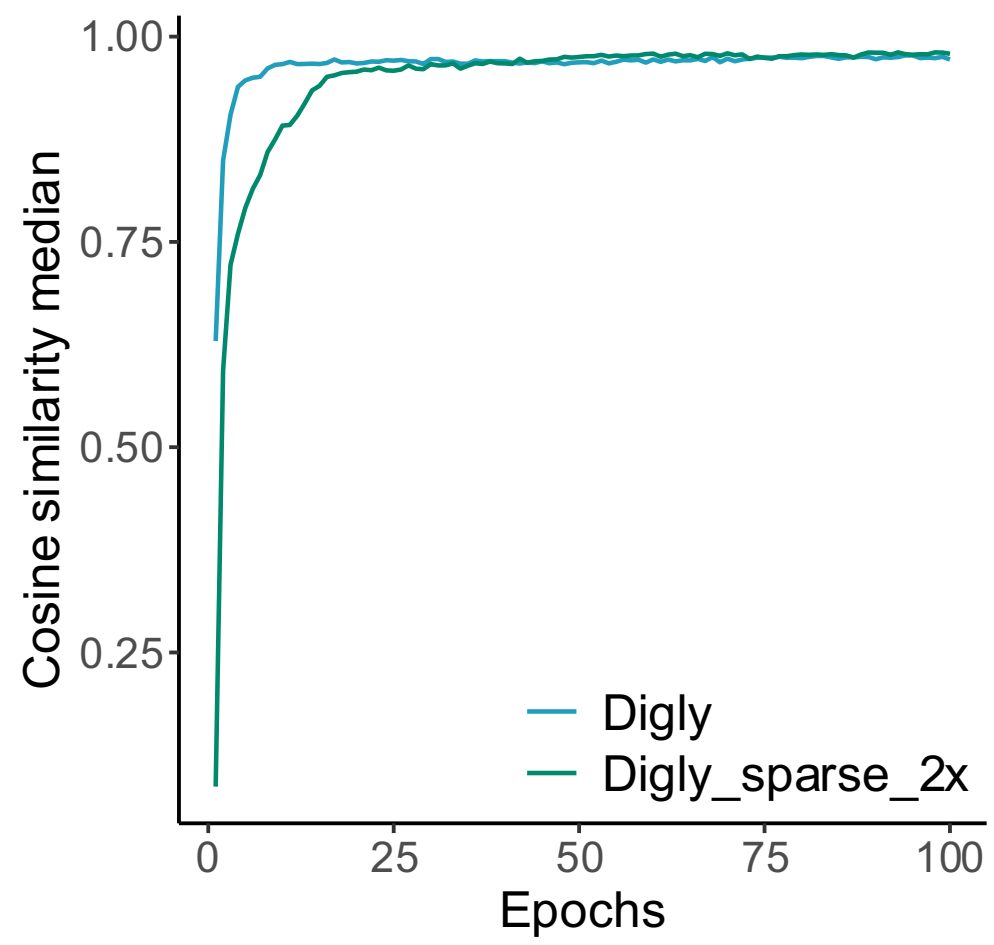

Figure S12. Performance evaluation of sparsity augmentation in DeepGPO training on the multiply glycopeptides dataset (Dataset 12). Digly: fragmentation types corresponding to di-glycosylated peptides are considered for all the MS/MS spectra (even the spectra identified as mono-glycosylated peptides) in Dataset 12. Digly sparse 2×: zero-padding is applied to the B/Y ion channels of all the MS/MS spectra in Dataset 12 to further enlarge the matrix (the final B/Y ion matrix size is 2 times of the original one).

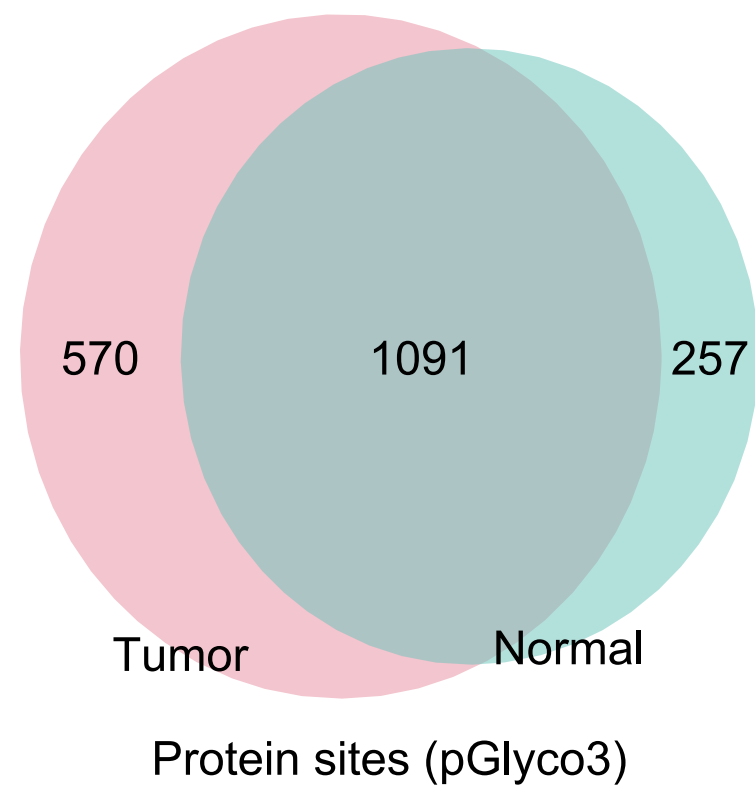

Figure S13. The Venn diagram shows shared and sample-specific *O*-glycosylation sites between kidney tumor and adjacent normal tissues by pGlyco3.

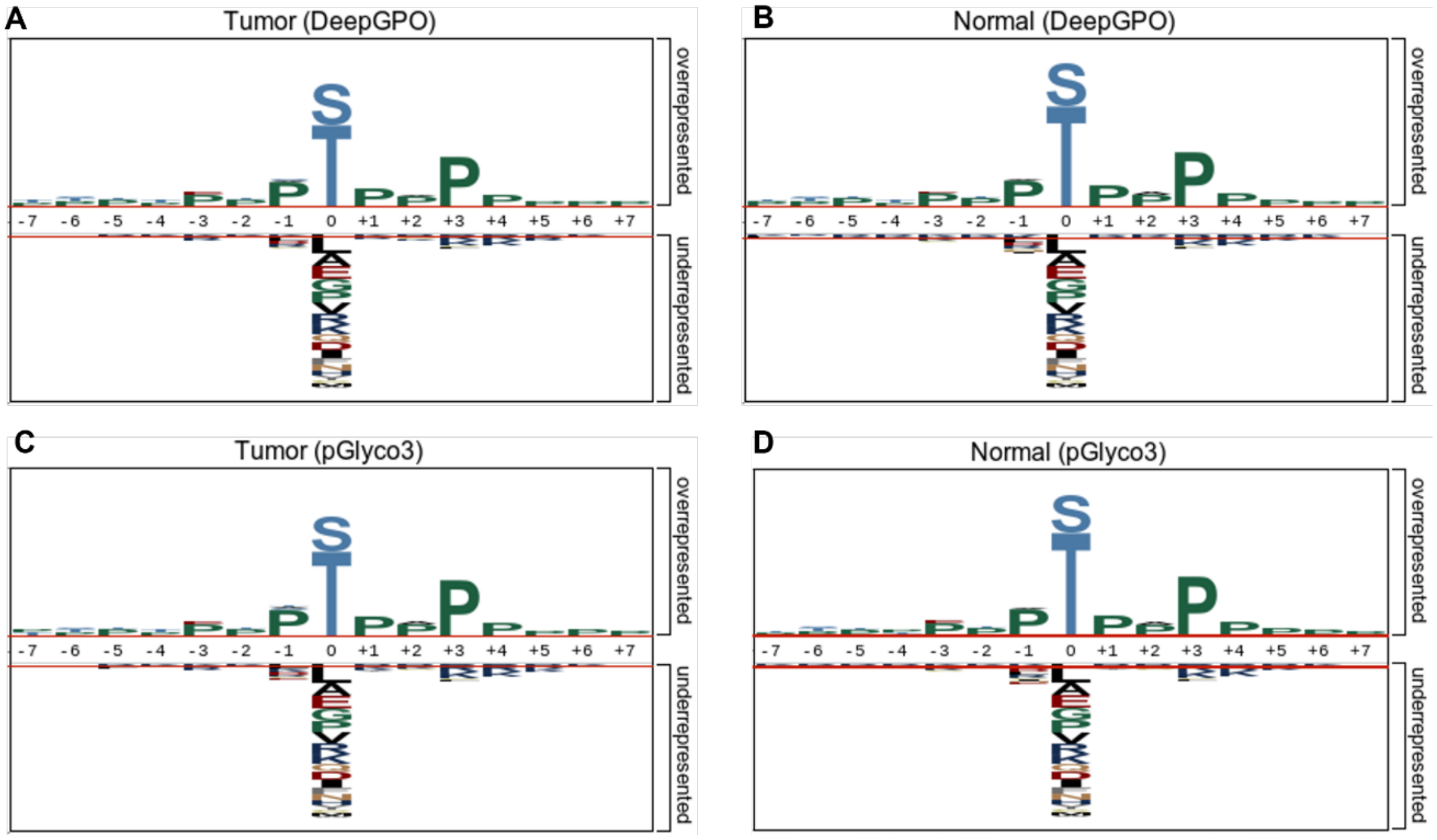

Figure S14. Analysis of amino acid sequence surrounding *O*-linked glycosylation sites for (**A**) tumor samples by DeepGPO, (**B**) adjacent normal tissues by DeepGPO, (**C**) tumor samples by pGlyco3 and (**D**) adjacent normal tissues by pGlyco3.

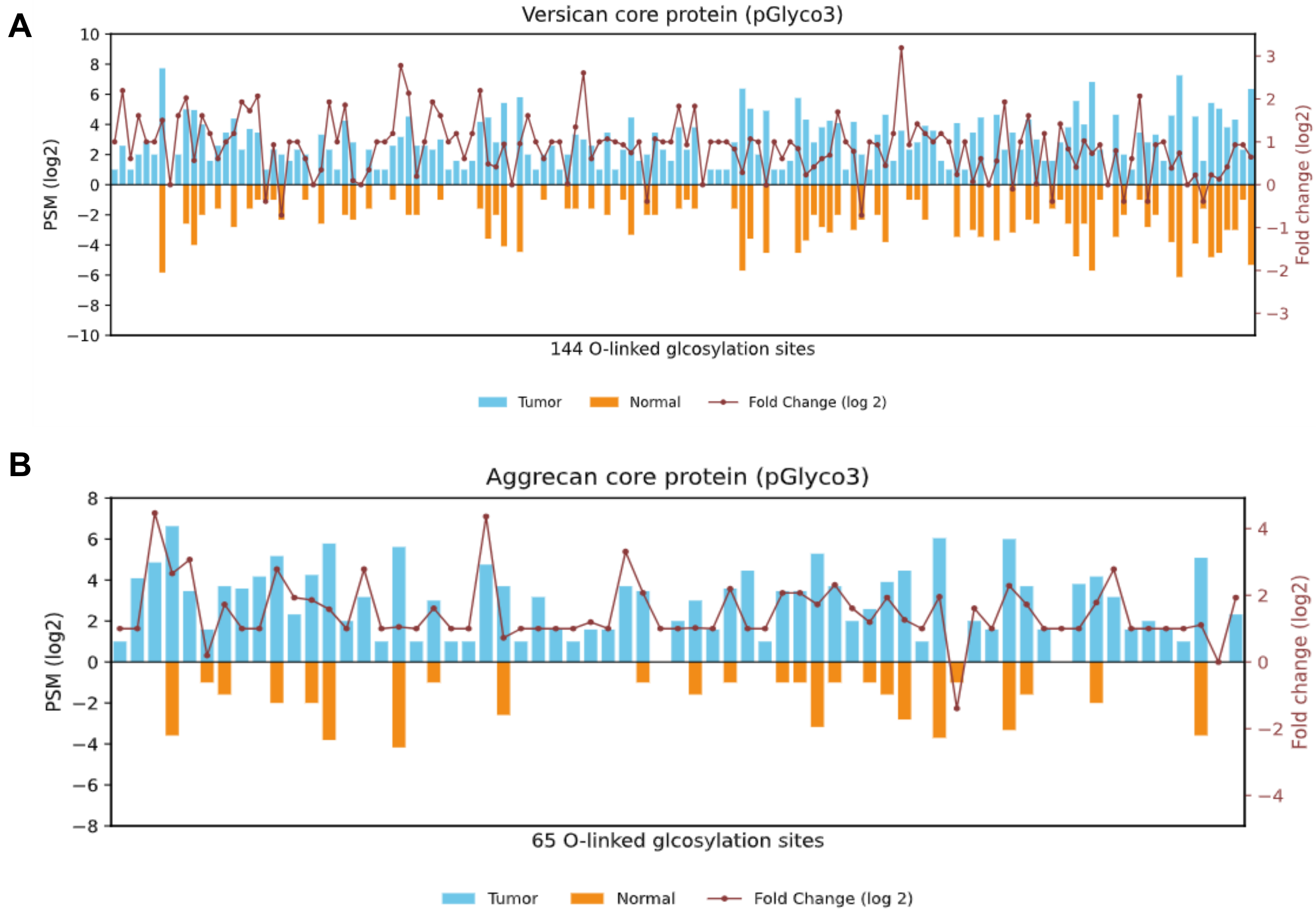

Figure S15. Site-level comparison of *O*-glycosylation patterns on **(A)** versican core protein (VCAN) and **(B)** aggrecan core protein (ACAN) in kidney tumor and adjacent normal tissues based on pGlyco3 site assignment.

Table S1. Datasets for deep learning model training, evaluation and test.

Name	Instrument	Sample type	*O*-glycoproteases	Accession
Dataset 1	Orbitrap Fusion Lumos Tribrid	Human / Mouse	IMPa/None	PXD037415[1]
Dataset 2	LTQ-Orbitrap Velos / Orbitrap Fusion Tribrid	Human / Pig	None	PXD018560[2]
Dataset 3	Orbitrap Fusion Tribrid	Human	None	PXD004590[3]
Dataset 4	Orbitrap Fusion Tribrid	Mucin	AM0627	PXD032164[4]
Dataset 5	Orbitrap Fusion Lumos Tribrid	SARS-CoV-2	None	PXD022896[7]
Dataset 6	Q-Exactive HF	Human	OgpA	PXD009476[5]
Dataset 7	Orbitrap Fusion Tribrid	Bovine / Human	OgpA	PXD020077[8]
Dataset 8	Orbitrap Eclipse Tribrid	T cell immunoglobulin and mucin-domain-containing (TIM) proteins	IMPa/OgpA/SmE/ None	PXD039583[9]
Dataset 9	Orbitrap Fusion Tribrid	Mucin	None	PXD027616[10]
Dataset 10	Orbitrap Fusion Tribrid	Mucin	AM0627	PXD031225[4]
Dataset 11	Orbitrap Fusion Tribrid	Mucin	IMPa/OgpA/StcE	PXD035775[11]
Dataset 12	Orbitrap Fusion	Mucin	StcE	PXD017646[6]

References

[1] S. Suttapitugsakul, Y. Matsumoto, R. P. Aryal, R. D. Cummings, "Large-Scale and Site-Specific Mapping of the Murine Brain O-Glycoproteome with IMPa," *Analytical Chemistry* 95 (2023): 13423. https://doi.org/10.1021/acs.analchem.3c00408

[2] T. D. Madsen, L. H. Hansen, J. Hintze, Z. Ye, S. Jebari, D. B. Andersen, H. J. Joshi, T. Ju, J. P. Goetze, C. Martin, M. M. Rosenkilde, J. J. Holst, R. E. Kuhre, C. K. Goth, S. Y. Vakhrushev, K. T. Schjoldager, "An atlas of O-linked glycosylation on peptide hormones reveals diverse biological roles," *Nature Communications* 11 (2020): 4033. https://doi.org/10.1038/s41467-020-17473-1

[3] S. L. King, H. J. Joshi, K. T. Schjoldager, A. Halim, T. D. Madsen, M. H. Dziegiel, A. Woetmann, S. Y. Vakhrushev, H. H. Wandall, "Characterizing the O-glycosylation landscape of human plasma, platelets, and endothelial cells," *Blood Advances* 1 (2017): 429. https://doi.org/10.1182/bloodadvances.2016002121

[4] D. J. Shon, D. Fernandez, N. M. Riley, M. J. Ferracane, C. R. Bertozzi, "Structure-guided mutagenesis of a mucin-selective metalloprotease from Akkermansia muciniphila alters substrate preferences," *Journal of Biological Chemistry* 298 (2022): 101917. https://doi.org/10.1016/j.jbc.2022.101917

[5] W. Yang, M. Ao, Y. Hu, Q. K. Li, H. Zhang, "Mapping the O-glycoproteome using site-specific extraction of O-linked glycopeptides (EXoO)," *Molecular Systems Biology* 14 (2018): e8486. https://doi.org/10.15252/msb.20188486

[6] N. M. Riley, S. A. Malaker, M. D. Driessen, C. R. Bertozzi, "Optimal Dissociation Methods Differ for N- and O-Glycopeptides," *Journal of Proteome Research* 19 (2020): 3286. https://doi.org/10.1021/acs.jproteome.0c00218

[7] Y. Zhang, W. J. Zhao, Y. H. Mao, Y. H. Chen, S. S. Zheng, W. Cao, J. Q. Zhu, L. Q. Hu, M. Gong, J. Q. Cheng, H. Yang, "O-Glycosylation Landscapes of SARS-CoV-2 Spike Proteins," *Frontiers in Chemistry* 9 (2021): 689521. https://doi.org/10.3389/fchem.2021.689521

[8] N. M. Riley, S. A. Malaker, C. R. Bertozzi, "Electron-Based Dissociation Is Needed for O-Glycopeptides Derived from OpeRATOR Proteolysis," *Analytical Chemistry* 92 (2020): 14878. https://doi.org/10.1021/acs.analchem.0c02950

[9] J. Chongsaritsinsuk, A. D. Steigmeyer, K. E. Mahoney, M. A. Rosenfeld, T. M. Lucas, C. M. Smith, A. Li, D. Ince, F. L. Kearns, A. S. Battison, M. A. Hollenhorst, D. Judy Shon, K. H. Tiemeyer, V. Attah, C. Kwon, C. R. Bertozzi, M. J. Ferracane, M. A. Lemmon, R. E. Amaro, S. A. Malaker, "Glycoproteomic landscape and structural dynamics of TIM family immune checkpoints enabled by mucinase SmE," *Nature Communications* 14 (2023): 6169. https://doi.org/10.1038/s41467-023-41756-y

[10] K. Pedram, N. N. Laqtom, D. J. Shon, A. Di Spiezio, N. M. Riley, P. Saftig, M. Abu-Remaileh, C. R. Bertozzi, "Lysosomal cathepsin D mediates endogenous mucin glycodomain catabolism in mammals," *Proceedings of the National Academy of Sciences of the United States of America* 119 (2022): e2117105119. https://doi.org/10.1073/pnas.2117105119

[11] N. M. Riley, C. R. Bertozzi, "Deciphering O-glycoprotease substrate preferences with O-Pair Search," *Molecular Omics* 18 10 (2022): 908. https://doi.org/10.1039/d2mo00244b
